# Endolymphatic Hydrops is a Marker of Synaptopathy Following Traumatic Noise Exposure

**DOI:** 10.3389/fcell.2021.747870

**Published:** 2021-11-05

**Authors:** Ido Badash, Patricia M. Quiñones, Kevin J. Oghalai, Juemei Wang, Christopher G. Lui, Frank Macias-Escriva, Brian E. Applegate, John S. Oghalai

**Affiliations:** ^1^ Caruso Department of Otolaryngology-Head and Neck Surgery, Keck School of Medicine of the University of Southern California, Los Angeles, CA, United States; ^2^ Viterbi School of Engineering, University of Southern California, Los Angeles, CA, United States; ^3^ Department of Otolaryngology-Head and Neck Surgery, Northwestern University Feinberg School of Medicine, Chicago, IL, United States

**Keywords:** hidden hearing loss, acoustic trauma, ribbon synapse, cochlear synaptopathy, endolymphatic hydrops

## Abstract

After acoustic trauma, there can be loss of synaptic connections between inner hair cells and auditory neurons in the cochlea, which may lead to hearing abnormalities including speech-in-noise difficulties, tinnitus, and hyperacusis. We have previously studied mice with blast-induced cochlear synaptopathy and found that they also developed a build-up of endolymph, termed endolymphatic hydrops. In this study, we used optical coherence tomography to measure endolymph volume in live CBA/CaJ mice exposed to various noise intensities. We quantified the number of synaptic ribbons and postsynaptic densities under the inner hair cells 1 week after noise exposure to determine if they correlated with acute changes in endolymph volume measured in the hours after the noise exposure. After 2 h of noise at an intensity of 95 dB SPL or below, both endolymph volume and synaptic counts remained normal. After exposure to 2 h of 100 dB SPL noise, mice developed endolymphatic hydrops and had reduced synaptic counts in the basal and middle regions of the cochlea. Furthermore, round-window application of hypertonic saline reduced the degree of endolymphatic hydrops that developed after 100 dB SPL noise exposure and partially prevented the reduction in synaptic counts in the cochlear base. Taken together, these results indicate that endolymphatic hydrops correlates with noise-induced cochlear synaptopathy, suggesting that these two pathologic findings have a common mechanistic basis.

## Introduction

Acoustic trauma is the most common preventable cause of hearing loss, and it has been suggested that 12% or more of the world population is at risk for noise-induced loss of hearing (Alberti et al., 1979; Le et al., 2017). Research in mice, guinea pigs, and rhesus macaques has shown that even moderate noise exposure levels previously thought to cause only temporary threshold shifts can result in immediate and irreversible loss of the synaptic connections between inner hair cells (IHCs) and cochlear nerve fibers (Kujawa and Liberman, 2009; Jensen et al., 2015; Kujawa and Liberman, 2015; Liberman et al., 2015; Valero et al., 2017). As most of the nerve fibers affected by this change have high thresholds and low spontaneous rates of firing, the loss of ribbon synapses does not elevate behavioral auditory thresholds or auditory brainstem response (ABR) thresholds until it becomes extreme. This phenomenon has thus been called hidden hearing loss, since it would not be detected on traditional hearing tests (Liberman et al., 2016). While some studies suggest that cochlear synaptopathy is not common in humans (Prendergast et al., 2017; Guest et al., 2019a; Guest et al., 2019b), other studies argue that it contributes to a variety of hearing abnormalities including speech-in-noise difficulties, tinnitus and hyperacusis (Felder and Schrott-Fischer, 1995; Roberts et al., 2010; Hickox and Liberman, 2014; Viana et al., 2015; Liberman and Kujawa, 2017).

Noise-induced damage to auditory nerve dendrites is caused by excess release of glutamate, the neurotransmitter responsible for afferent signaling between hair cells and auditory neurons (Spoendlin, 1971; Robertson, 1983; Choi and Rothman, 1990; Pujol et al., 1993; Puel et al., 1998; Kim et al., 2019a). We have previously studied mice exposed to blast pressure waves and found widespread cochlear synaptopathy 1 week after the blast (Kim et al., 2018a). Using optical coherence tomography (OCT) to image the mouse cochlea non-invasively right after the blast, we also identified a build-up of fluid within the scala media, known as endolymphatic hydrops. Interestingly, treating the endolymphatic hydrops with hypertonic saline also reduced cochlear synaptopathy. Thus, although these data do not prove that endolymphatic hydrops causes cochlear synaptopathy, they suggest that they may be related.

Here, we sought to better assess this relationship by using a non-blast noise exposure protocol that permitted us to better titrate the level of the acoustic trauma. We examined the relationship between noise intensity, endolymph volume, and synapse loss, and found that endolymphatic hydrops correlates with the loss of IHC ribbons and postsynaptic densities (PSDs). Moreover, we showed that noise-induced endolymphatic hydrops and loss of cochlear synapses could be mitigated through the round window application of hypertonic saline, further suggesting that these two processes have a common mechanistic basis.

## Materials and Methods

### Animals

All experiments were performed according to protocols approved by the Institutional Animal Care and Use Committee at the University of Southern California. We used a total of 59 CBA/CaJ mice that were 4- to 6-weeks old. Anesthesia consisted of a combination of ketamine (100 mg/kg) and xylazine (10 mg/kg).

### Noise Exposure

Our noise exposure protocol has been previously published (Liu et al., 2011; Xia et al., 2013). Briefly, awake mice were placed inside a plastic cage with custom-built subdivisions made from chicken wire, such that each animal had its own area to freely move about. This allowed exposure of up to four mice simultaneously. The cage was fitted with a roof also made from chicken wire and placed inside a wooden box with speakers built into the lid. White noise that was bandpass filtered between 8 and 16 kHz was delivered to the mice for 2 h. The noise intensity was monitored using a ¼″ Brüel & Kjaer microphone and was consistent throughout the cage and over the course of the 2 h exposure within the range of ±2 decibel (dB) sound pressure level (SPL). Mice designated as controls were placed in the exact same experimental environment as the noise-exposed mice for 2 h but without noise delivered through the speakers.

### *In Vivo* OCT Imaging

After noise or sham exposure, 21 of the 59 mice underwent cochlear imaging to measure endolymph volume, as previously described (Kim et al., 2018a). Anesthetized mice were positioned on a heating pad to maintain a core body temperature of 37°C, and additional doses of anesthesia were administered throughout the experiment to maintain sedation. The skull was exposed and glued to a head-holder with dental cement. A ventrolateral approach was used to surgically access the left middle ear bulla, which was opened carefully by microdissection to access the apical turn of the cochlea without disturbing the otic capsule. Our custom-built OCT system has been previously described (Dewey et al., 2019). Two-dimensional imaging of the cochlear duct was performed by repeatedly scanning the optical beam to collect cross-sectional images in the x and z dimensions. To quantify endolymph volume, we collected a volume stack of cross-sectional images of the cochlea, moving the y position in 2 μm steps over a 300-μm length of the basement membrane (150 cross-sections per mouse). The orientation of the mouse cochlea from our surgical approach and angle of the OCT scanning laser allowed us to image a limited portion of the apical turn centered at the 9 kHz location, which we know from previous studies in which we measured vibratory tuning curves at this cochlear position (Gao et al., 2014; Lee et al., 2015; Lee et al., 2016; Dewey et al., 2018; Dewey et al., 2019). We were able to collect images along an approximately 300-μm length of the cochlear duct at this location, and then used Imaris software (Bitplane, Concord, MA) to render 3D images from this volume stack. We removed 75-μm segments on both ends of the volume stack to select an identical 150-μm segment of the scala media from each sample, as we have previously reported (Kim et al., 2018a). The volume of this 150-μm long chamber was measured though a built-in feature within Imaris using a calculated voxel size based on the scanning parameters of the laser. A cochleogram showing the location of the 300-μm region where OCT was used to image the cochlea, and the 150-μm subsection of this region where endolymph volume of the scala media was measured, is included in Supplementary Figure S1.

### Immunofluorescence and Cochlear Dissection

Thirty-eight of the 59 mice were assessed for cochlear synaptopathy. Following noise or sham exposure, these mice were returned to the animal facility for routine care. Our methods of immunofluorescence have been previously reported (Kim et al., 2018a). One week after noise or sham exposure, the mice were euthanized with isofluorane and both cochleae were extracted. We opened a fenestra in the apex and perfused 4% paraformaldehyde through the round window. Following this, we immersed the cochlea in 4% paraformaldehyde solution at room temperature for 30 min. After washing with PBS, the cochlea was decalcified by immersing in a 0.5 M EDTA solution (pH 8) for 6 h at room temperature, and again washed in PBS.

The sensory epithelium was then dissected into apical, middle, and basal sections in a manner similar to the whole mount dissection technique reported by Montgomery and Cox (2016). The average lengths and variances of these segments were measured and converted into percentages of the total cochlear length. These percentages were correlated with their respective tonotopic frequencies based on the cochlear place-frequency maps described by Müller et al. (2005) and Viberg and Canlon (2004). The apical and middle segments each measured 1.9 ± 0.1 mm (mean ± standard error), and the basal segment measured 1.8 ± 0.05 mm. Approximately 10 ± 5% of the cochlea, corresponding to the hook region, was damaged due to limitations of the dissection and not included in our analysis. Assuming the CBA/CaJ cochlea ranges from 5 kHz at the apex to 80 kHz at the base (Viberg and Canlon, 2004; Müller et al., 2005), then the apical segment corresponds to the frequency range of 5 − 11.5 ± 0.5 kHz, the middle segment corresponds to the frequency range of 11.5 ± 0.5 − 26 ± 2 kHz, and the base segment corresponds to the frequency range of 26 ± 2 − 60 ± 8 kHz. The average tonotopic frequencies and percentages of total cochlear length from the base associated with the apical, middle and base segments are shown in the cochleogram in Supplementary Figure S1.

Dissected cochlear tissues were incubated in blocking solution (5% donkey serum, 0.1% Triton X-100, and 1.0% BSA in PBS) for 1 h at room temperature. Samples were incubated with primary antibodies diluted in the same blocking solution for 2 days at 4°C followed by a 2 h incubation at 37°C. The primary antibody solution contained mouse anti-CtBP2 IgG (1:200; 612044 (Lot: 8172904), BD Biosciences) and rabbit anti-Homer IgG (1:800; 160003 (Lot: 1–43), Synaptic Systems). After washing in PBST (0.1% Triton X-100 in PBS) the tissues were incubated with a secondary antibody solution diluted in 0.1% Triton X-100 and 0.1% BSA in PBS for 1 h at room temperature. The secondary antibodies were donkey anti-mouse IgG conjugated with Alexa Fluor 488 (1:500; A21202, Invitrogen) and donkey anti-rabbit IgG conjugated with Alexa Fluor 546 (1:500; A10040, Invitrogen). Alexa Fluor 647-conjugated phalloidin (1:200; A22287, Invitrogen) was added with the secondary antibody solution. After washing in PBS, the tissues were mounted on glass slides using Fluoromount-G with DAPI (00-4959-52, Invitrogen). Slides were kept overnight at 4°C before imaging with an upright confocal microscope (Zeiss LSM 800) using a 63X objective (1.4 N.A.) to generate z-stacks.

### Identification and Co-Localization of synaptic Ribbons and Postsynaptic Densities

The number of IHCs, outer hair cells (OHCs), CtBP2-labeled synaptic ribbons, and Homer-labeled PSDs were counted using custom image processing software written in MATLAB (R2021a, The MathWorks Inc., Natick, MA). The number of IHCs and OHCs were counted manually by visual inspection of hair cell nuclei in each z-stack. Automated counts of ribbons and PSDs were then performed (Supplementary Figure S2). First, the background was developed by running the images through a 1.4 μm median filter to remove all objects at or below the size of ribbons and PSDs. The original images were passed through a 0.35 μ median filter to eliminate speckle noise, and the background was subtracted from these images to isolate the ribbon- and PSD-sized objects. Ribbons and PSDs were identified by picking only those objects that were more intense than a threshold level selected by eye. The program then grouped these objects by X, Y, and Z coordinates, with each group signifying a single ribbon or density. Structures which were shorter than 0.48 µm were deemed not tall enough to be ribbons or PSDs and were removed by the program. Co-localization of ribbons and PSDs was performed using the X, Y, and Z coordinates of these structures, such that nearby structures within 2 μm of each other were identified as a pair. Ribbons that were not paired with a corresponding PSD were determined to be orphan ribbons. All counts were verified and adjusted based on visual inspection by two blinded investigators acting independently. If there were any discrepancies between the final counts of the two independent reviewers, these were resolved by the senior author after independent, blinded review.

### Application of Solutions to the Round Window Membrane

Of the 24 mice undergoing *in vivo* OCT imaging after noise or sham exposure, in 6 mice we surgically opened the middle ear bulla under anesthesia as described above and applied either hypertonic saline (6,000 mOsm/kg) or normotonic saline (307 mOsm/kg) to fill the bulla and cover the round window membrane. The solution was drawn up using a rolled Kimwipe and re-applied every 15 min in order to maintain the desired osmolality. The solution was also withdrawn and re-applied every time an image or volume stack was captured using OCT.

Thirteen of the 38 mice that were to be used for immunolabeling experiments were anesthetized immediately following noise or sham exposure and underwent round-window application of either hypertonic or normotonic saline. We pierced a small hole in the tympanic membrane under microscopic guidance and filled the middle ear space with the test solution through the perforation until fluid appeared in the ear canal. Only left ears were treated. Mice were maintained under anesthesia for 5.5 h from the time of intratympanic injection while lying with the left ear up to keep the test solution in contact with the round window membrane. Additional test solution was instilled into the ear every 15 min in order to maintain the desired osmolality.

Normotonic saline (307 mOsm/kg) was composed of 150 mM NaCl and 20 mM HEPES. Hypertonic saline (6,000 mOsm/kg) was composed of 2,990 mM NaCl and 20 mM HEPES. For both solutions, the pH was adjusted to 7.4 using a benchtop pH meter and either 1 M NaOH or 1 M HCl. The osmolality of normotonic saline was verified using a freezing pressure osmometer (3,320, Advanced Instruments). The osmolality of the hypertonic solution could only be predicted based on its constituents, as its osmolality exceeded the upper threshold of the osmometer (2000 mOsm/kg).

### Statistical Analysis

Statistical analysis and data plotting was performed using GraphPad Prism (version 8.0.2, GraphPad Software Inc., La Jolla, CA). All data sets were tested for the presence of a normal distribution using the Shapiro-Wilk test for normality. Changes in endolymph volume across time between different noise intensities and treatment conditions were compared using repeated measures two-way ANOVA with the Geisser-Greenhouse correction and post-hoc Tukey multiple comparisons test. Ribbons, PSDs, and orphan ribbon counts in the base, middle and apex of the cochlea were compared between different noise intensities and treatment conditions using two-way ANOVA with post-hoc Tukey multiple comparisons test. Sum of squares calculations were performed as part of the two-way ANOVA to correct for imbalances caused by unequal sample sizes among groups (Landsheer and van den Wittenboer, 2015; Glantz et al., 2016; GraphPad Statistics Guide, 2021). All tests were two tailed, and a *p* value of <0.05 was considered statistically significant. In cases where the *p* values calculated from two-way ANOVA were statistically significant, only the *p* values for single-pair comparisons from the post-hoc Tukey multiple comparisons test are reported. All means are presented with standard errors and sample sizes. The results of all statistical tests performed in this study are provided in Supplementary Tables S1–9.

## Results

### 100 dB SPL 2-h Noise Exposure Produces Endolymphatic Hydrops

**FIGURE 1:**
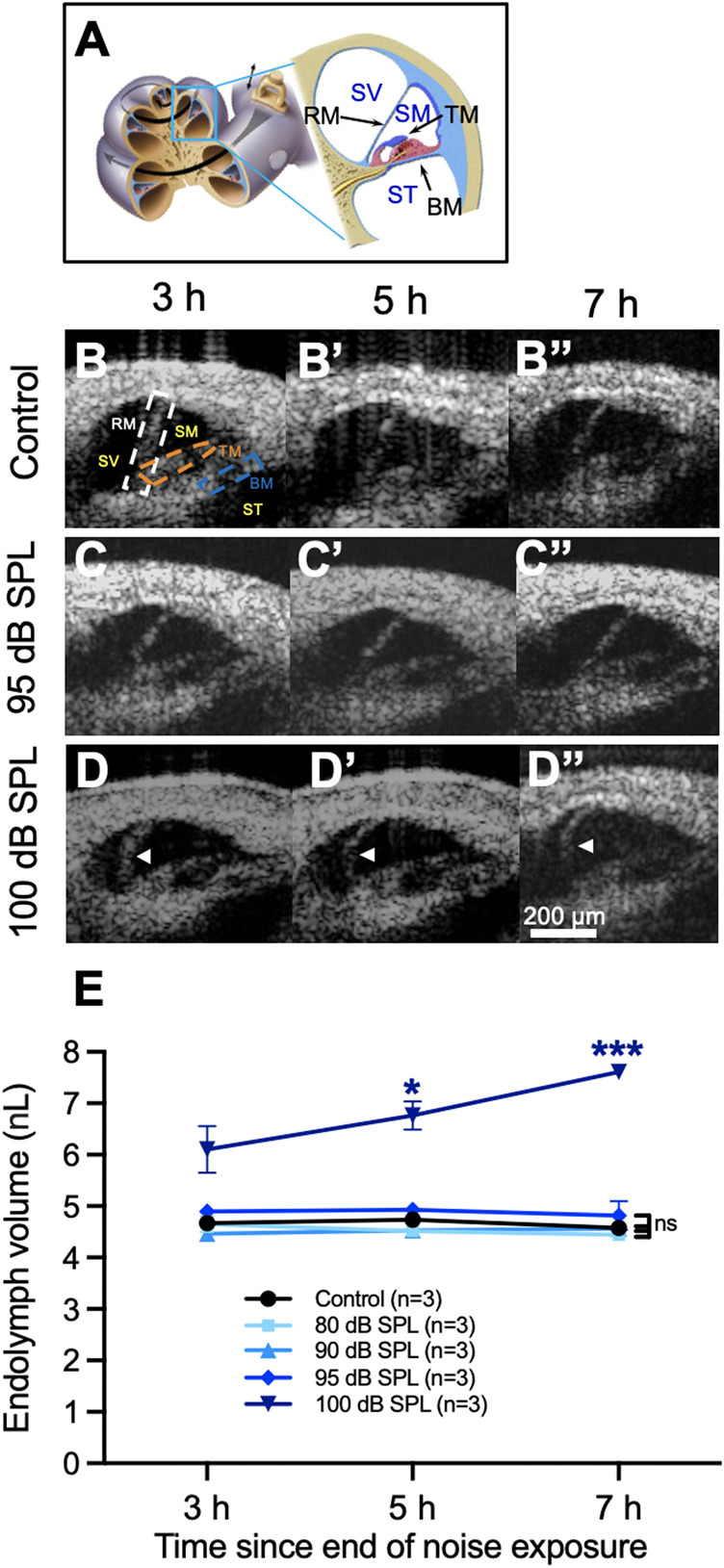
Endolymphatic hydrops develops after 2 h of 100 decibel (dB) sound pressure level (SPL) noise exposure. **(A)** Diagram of the mouse cochlea indicating the location where optical coherence tomography (OCT) was performed within the apical turn. Locations of Reissner’s membrane (RM), basilar membrane (BM), tectorial membrane (TM), scala vestibuli (SV), scala media (SM) and scala tympani (ST) are shown. **(B)** OCT images from a representative control mouse demonstrate normal endolymph volume and no change in the position of RM over time. Locations of the same structures from panel **(A)** are shown in the first panel. **(C)** OCT images from a mouse exposed to 95 dB SPL noise also demonstrate normal endolymph volume and no bulging of RM over time. **(D)** Endolymphatic hydrops is apparent at 3 h following 100 dB SPL noise exposure and progressively grows over the next 4 h. White arrow heads indicate the bowed position of RM. **(E)** Quantification of endolymph volume measured over time in mice exposed to 80, 90, 95, and 100 dB SPL as well as control mice that were not exposed to noise. Endolymph volume increased over time in mice exposed to 100 dB SPL and was significantly greater than control mice at 5 and 7 h following noise exposure. Endolymph volumes in mice exposed to lower noise intensity levels were not significantly different than in control mice. Endolymph volume represents the volume of the scala media over a segment of basement membrane that is 150 μm in length. Sample sizes are expressed in number of mice. Error bars indicate standard error. *p < 0.05, ***p < 0.001.

First, we titrated the level of acoustic trauma to determine the threshold for developing endolymphatic hydrops. We subjected cohorts of mice to 2 h of sham exposure (control, no noise) or noise exposure at an intensity of 80, 90, 95, or 100 dB SPL and serially imaged the apical turn of the cochlea *in vivo* using OCT. Reissner’s membrane (RM), the basilar membrane (BM), and the tectorial membrane (TM) could all be resolved in the resulting cross-sectional image of the cochlea (Figures 1A,B). We anesthetized mice 2 h after the noise exposure was completed and used ∼1 h to dissect and prepare the mouse. We then imaged the apical turn of the cochlea from 3–7 h after the noise exposure. Thus, the total anesthetic time was limited to 5 h.

In unexposed control mice and those exposed to noise intensity levels of 80, 90, or 95 dB SPL, there were no changes in the position of RM over time (Figures 1B,C, Supplementary Movie S1). In contrast, mice exposed to 100 dB SPL noise had progressive bulging of RM over time consistent with an increase in endolymph volume, termed endolymphatic hydrops (Figure 1D, Supplementary Movie S2).

We then quantified endolymph volume in these cohorts of mice (Figure 1E). Endolymph volume increased by 24.6 ± 7.8% between 3 and 7 h after noise exposure in mice exposed to 100 dB SPL, while no such increase was observed in unexposed control mice or those exposed to 80, 90, or 95 dB SPL noise. Furthermore, endolymph volume was significantly greater in mice exposed to 100 dB SPL compared with unexposed control mice at 5 h (6.8 ± 0.3 nL, *n* = 3 vs. 4.7 ± 0.04 nL, *n* = 3, *p* = 0.0496) and 7 h (7.6 ± 0.1 nL, *n* = 3 vs. 4.6 ± 0.1 nL, *n* = 3, *p* = 0.0006) following noise exposure. By contrast, there were no significant differences in endolymph volume between control mice and those exposed to noise intensities lower than 100 dB SPL at any time point (Supplementary Table S1).

### 100 dB SPL 2-h Noise Exposure Induces Inner Hair Cell Cochlear Synaptopathy

**FIGURE 2:**
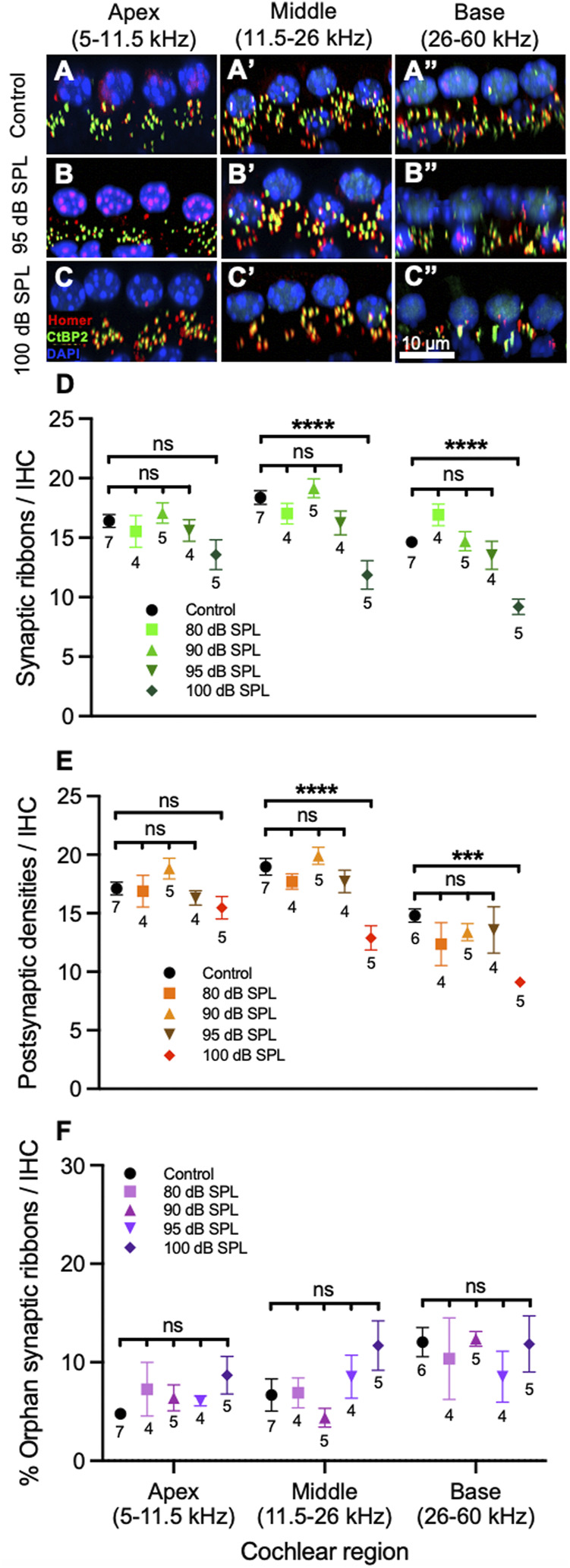
Sound intensity affects degree of inner hair cell (IHC) synapse loss following 2 h noise exposure. **(A–C)** Representative sections from the organ of Corti of mice 7 days after noise or sham exposure (control) displaying 4 IHC nuclei and associated ribbons as well as postsynaptic densities (PSDs). Immunolabeling was performed to visualize IHC ribbons (CtBP2, green), PSDs (Homer, red) and nuclei (DAPI, blue). Control mice **(A)** had a similar number of ribbons and PSDs when compared to mice exposed to 95 decibel (dB) sound pressure level (SPL) **(B)**. **(C)** A reduction in the number of ribbons and PSDs can be seen in the middle and basal cochlear regions of mice exposed to 100 dB SPL. **(D)** Quantification of ribbons per IHC. Mice exposed to 100 dB SPL noise had reduced numbers of ribbons per IHC in the middle and base of the cochlea when compared with control, unexposed mice. There were no significant differences in ribbons per IHC between control mice and those exposed to 80, 90, or 95 dB SPL in any region of the cochlea. **(E)** Quantification of PSDs per IHC. Mice exposed to 100 dB SPL had reduced numbers of PSDs per IHC in the middle and base of the cochlea when compared with control, unexposed mice. There were no significant differences in PSDs per IHC between control mice and those exposed to 80, 90, or 95 dB SPL in any region of the cochlea. **(F)** Comparison of the percentage of orphan ribbons (without an associated PSD) per IHC between mice exposed to different noise intensities. There were no significant differences in the percentage of orphan ribbons per IHC between control mice and those exposed to 80, 90, 95 or 100 dB SPL in any region of the cochlea. Data points represent means and error bars indicate standard error. Sample sizes are displayed under each data point and expressed in number of mice. All ribbon, PSD, and percentage of orphan ribbon counts per IHC represent averages of both the right and left ears when available. ns = not significant, ***p < 0.001, ****p < 0.0001.

To assess for cochlear synaptopathy, we counted the number of ribbons, PSDs, and percentage of orphan ribbons per IHC 7 days after noise exposure. This was done by immunolabeling for CtBP2, a marker for the presynaptic hair cell ribbon, Homer, a PSD scaffold protein, and DAPI, a counterstain for nuclear DNA (Figure 2). On gross visual inspection, control mice had a similar number of ribbons and PSDs per IHC when compared to mice exposed to 95 dB SPL throughout the cochlea (Figures 2A,B), while a reduction in the number of these structures was seen in the middle and basal turns of the cochlea in mice exposed to 100 dB SPL (Figure 2C).

**FIGURE 3:**
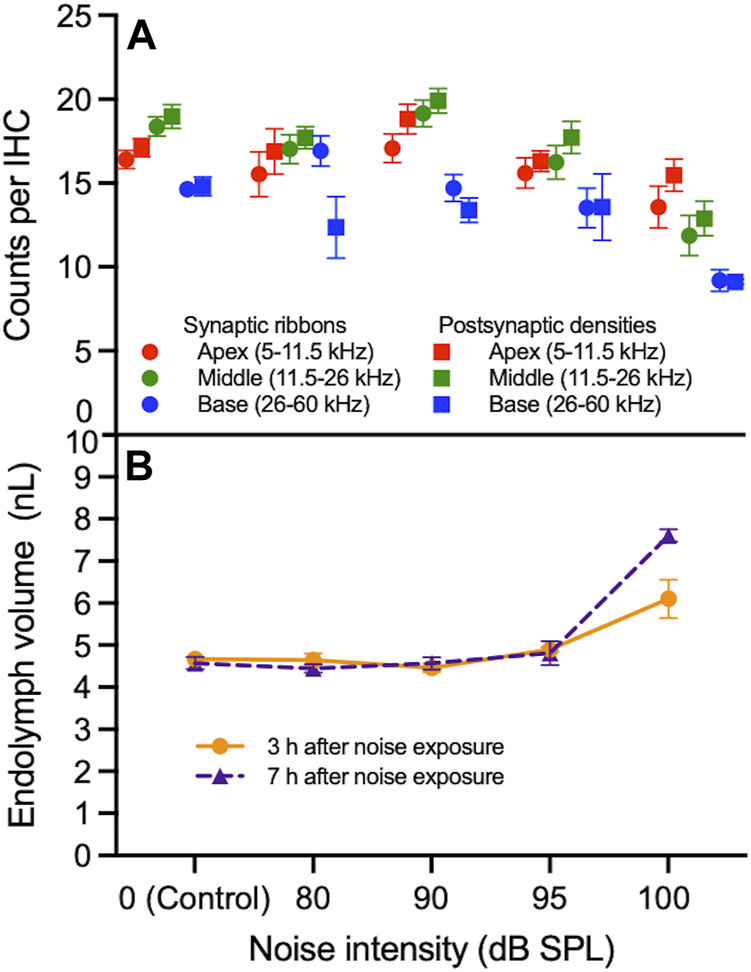
Correlation between synapse loss and endolymphatic hydrops as a function of noise intensity. **(A)** The numbers of synaptic ribbons and postsynaptic densities (PSDs) per inner hair cell (IHC) in the apex, middle and base of the cochlea do not progressively decrease between 0 (control) and 95 decibel (dB) sound pressure level (SPL) noise exposure. Between 95 and 100 dB SPL noise exposure, ribbons and PSDs per IHC decrease in the middle and base of the cochlea more sharply than in the apex. **(B)** Endolymph volume is relatively stable between 0 and 95 dB SPL at 3 and 7 h after noise exposure. Between 95 and 100 dB SPL noise exposure, endolymph volume increases; this increase is sharpest 7 h after noise exposure. Data points represent means and error bars indicate standard error. All ribbon and PSD counts per IHC represent averages of both the right and left ears when available. Endolymph volume represents the volume of the scala media over a segment of basement membrane that is 150 μm in length.

Next, we quantified the synaptic ribbons, PSDs, and percentage of orphan ribbons per IHC (Figures 2D–F). In the middle of the cochlea, the number of ribbons and PSDs per IHC in mice exposed to 100 dB SPL noise were 11.9 ± 1.2, *n* = 5 and 12.9 ± 1.0, *n* = 5, respectively, compared with 18.4 ± 0.6 ribbons, *n* = 7 and 19.0 ± 0.7 PSDs, *n* = 7 in unexposed control mice (*p* < 0.0001 for both ribbons and PSDs per IHC). By contrast, there were no significant differences in the numbers of ribbons or PSDs per IHC between control mice and those exposed to lower noise intensity levels (Supplementary Tables S2, 3). In the base of the cochlea, there were also significant reductions in the number of ribbons (9.2 ± 0.7, *n* = 5) and PSDs (9.1 ± 0.1, *n* = 5) per IHC in mice exposed to 100 dB SPL compared with control mice (14.6 ± 0.5 ribbons, *n* = 7 and 14.8 ± 0.6 PSDs, *n* = 6; *p* < 0.0001 for ribbons and *p* = 0.0002 for PSDs per IHC). Ribbons and PSDs per IHC in the base of the cochlea did not differ significantly between control mice and those exposed to lower noise intensities. In the cochlear apex, there were no significant differences in ribbons or PSDs per IHC between mice exposed to any noise intensity level and controls. Figure 3 displays the inverse relationship between endolymph volume and ribbon synapses in the middle and base of the cochlea as noise intensity increases. Of note, the percentage of orphan ribbons per IHC did not significantly differ between unexposed control mice and those exposed to any noise intensity level, including 100 dB SPL, in any region of the cochlea (Supplementary Table S4).

### 100 dB SPL Noise Exposure Does Not Cause Synaptopathy in Outer Hair Cells

**FIGURE 4:**
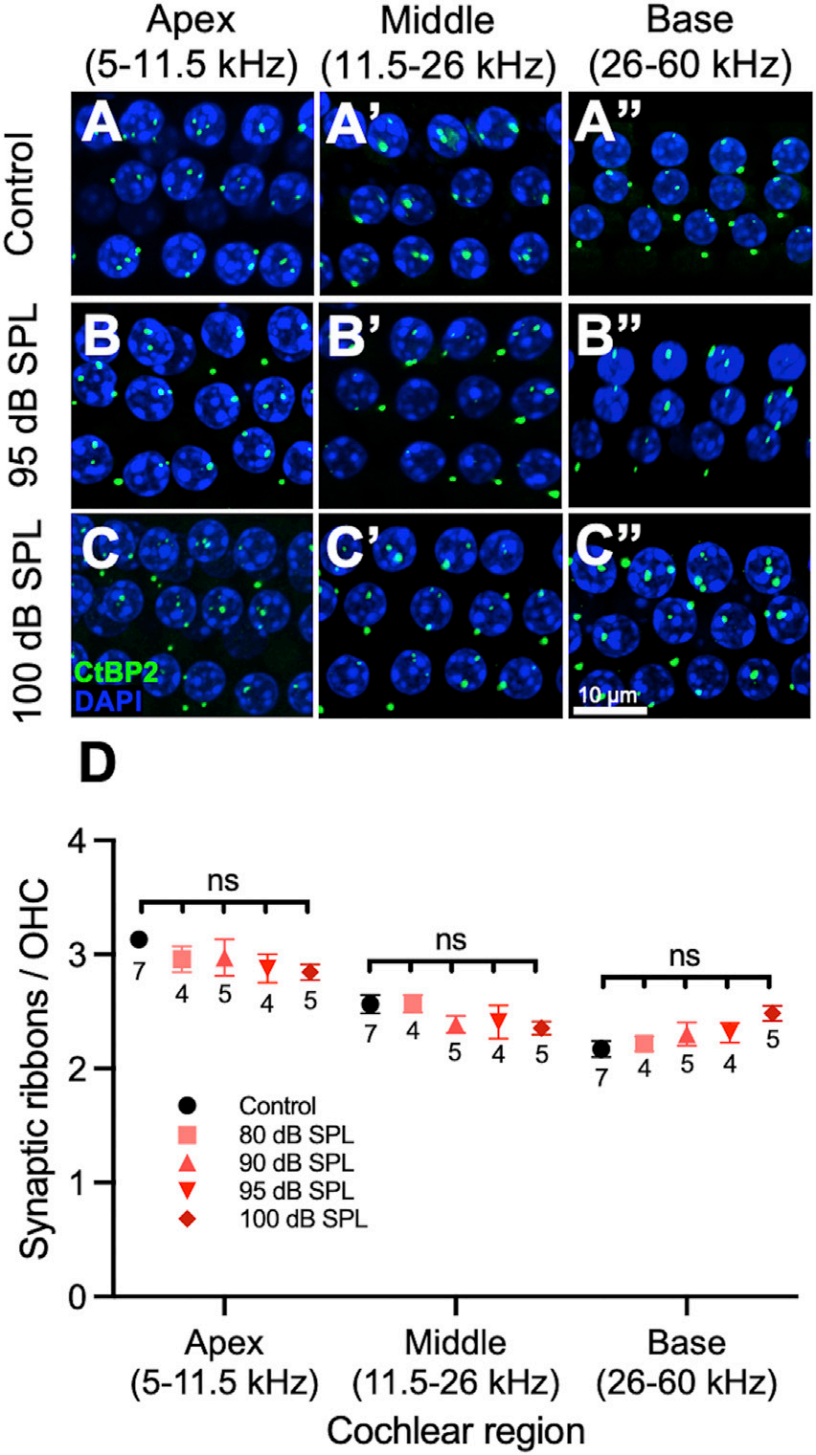
2 h noise exposure has no effect on outer hair cell (OHC) ribbons. **(A–C)** Representative sections from the organ of Corti of mice 7 days after noise or sham exposure (control) displaying 3 rows of 4 OHC nuclei and associated ribbons. Immunolabeling was performed to visualize OHC ribbons (CtBP2, green) as well as nuclei (DAPI, blue). Control mice **(A)**, mice exposed to 95 decibel (dB) sound pressure level SPL **(B)**, and mice exposed to 100 dB SPL **(C)** had similar numbers of ribbons per OHC. **(D)** Quantification of ribbons per OHC. There were no significant differences in the number of ribbons per OHC between mice exposed to 80, 90, 95, 100 dB SPL and control mice in any region of the cochlea. Data points represent means and error bars indicate standard error. Sample sizes are displayed under each data point and expressed in number of mice. All ribbon counts per OHC represent averages of both the right and left ears when available. ns = not significant.

We also counted synaptic ribbons in OHCs 7 days after noise exposure (Figure 4). There were no significant differences in ribbons per OHC between mice exposed to any noise intensity level and unexposed control mice in the apex, middle or base of the cochlea (Supplementary Table S5). PSDs were not assessed in OHC, since prior studies have shown that approximately half of Homer-immunolabeled PSDs in the OHC region are not associated with ribbons and would therefore not correlate with the presence of noise-induced synaptopathy (Martinez-Monedero et al., 2016).

### Round Window Application of Hypertonic Saline Reduces Endolymphatic Hydrops

We have previously shown that round window application of a hypertonic solution reduces endolymph volume, whereas hypotonic solutions increase it (Kim et al., 2018a). Here, we tested whether the application of hypertonic saline reduces endolymph volume following 100 dB SPL 2-h noise exposure.

**FIGURE 5:**
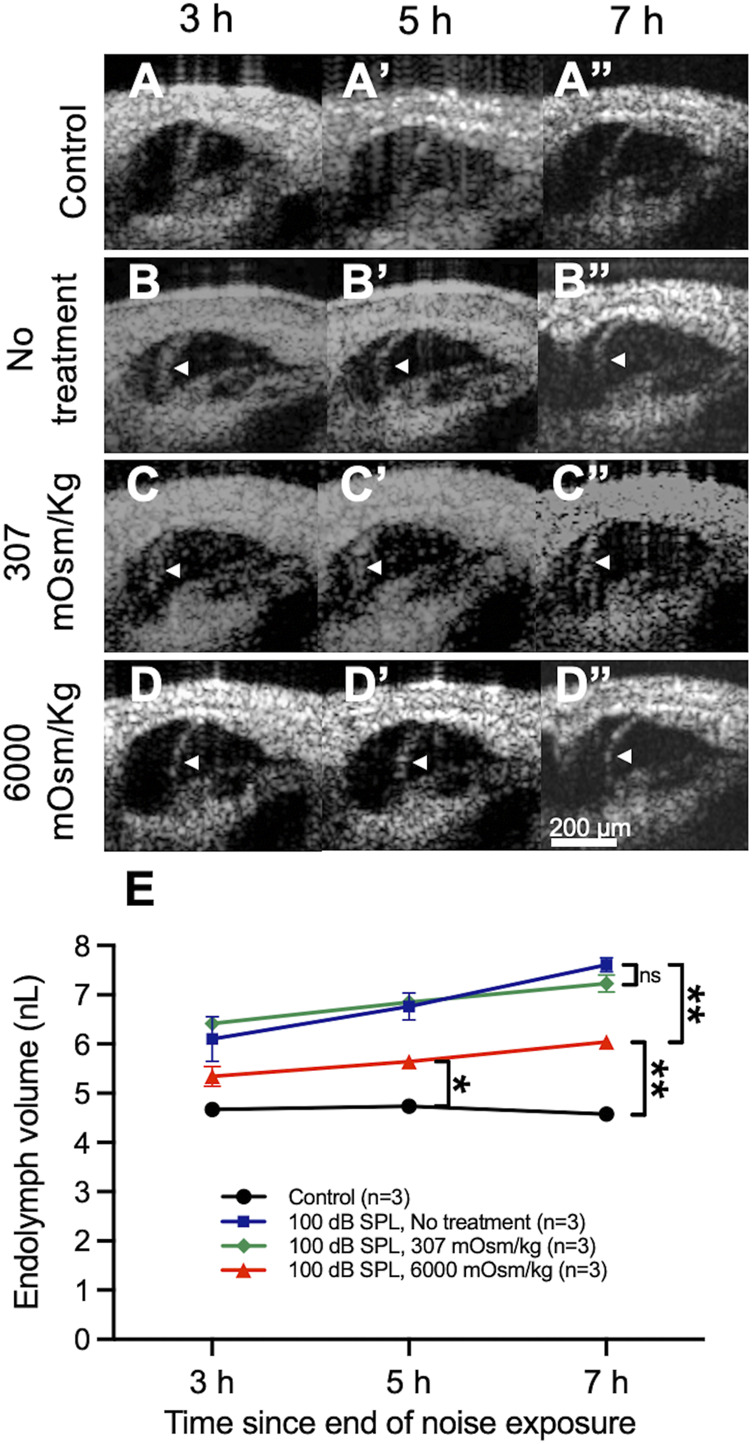
Osmotic treatment partially reduces the degree of endolymphatic hydrops that develops after 2 h of 100 decibel (dB) sound pressure level (SPL) noise exposure. **(A)** Control mice that were not exposed to noise had no change in endolymph volume over time and demonstrated no bowing of Reissner’s membrane (RM). **(B)** Noise-exposed mice without round window solution application (no treatment) developed posttraumatic endolymphatic hydrops with outward bowing of RM. White arrow heads indicate the bowed position of RM. **(C)** Round window application of normotonic saline (307 mOsm/kg) had no impact on endolymphatic hydrops. **(D)** Noise-exposed mice treated with hypertonic saline (6,000 mOsm/kg) showed a reduction of endolymphatic hydrops with decreased bowing of RM toward the scala vestibuli when compared with noise-exposed mice that were untreated or those treated with normotonic saline. **(E)** Quantification of endolymph volume over time in mice exposed to 100 dB SPL noise after undergoing round window application of hypertonic saline, normotonic saline, or no treatment, as well as unexposed control mice. Endolymph volume in noise-exposed mice treated with hypertonic saline was reduced compared with untreated mice and elevated compared with unexposed control mice at 7 h following the end of noise exposure. Endolymph volume in noise-exposed mice treated with normotonic saline was not significantly different than in noise-exposed untreated mice at any time point. Endolymph volume represents the volume of the scala media over a segment of basement membrane that is 150 μm in length. Sample sizes are expressed in number of mice. Error bars indicate standard error. ns = not significant, *p < 0.05, **p < 0.01.

Control mice that were not exposed to noise demonstrated no bowing of RM over time (Figure 5A), whereas mice exposed to 100 dB SPL without round window solution application developed posttraumatic endolymphatic hydrops with outward bowing of RM (Figure 5B). Round window application of normotonic saline (307 mOsm/kg) had no impact on endolymphatic hydrops when compared with untreated mice (Figure 5C), while noise-exposed mice treated with hypertonic saline (6,000 mOsm/kg) showed a reduction of endolymphatic hydrops with decreased outward bowing of RM toward the scala vestibuli when compared with noise-exposed mice that were untreated or those treated with normotonic saline (Figure 5D).

Quantification of endolymph volume for each treatment condition was then performed (Figure 5E). Endolymph volume increased by 13.0 ± 4.4% between 3 and 7 h after noise exposure in mice treated with hypertonic saline after exposure to 100 dB SPL. Mice treated with hypertonic saline had significantly reduced endolymph volume 7 h after 100 dB SPL noise exposure (6.0 ± 0.1 nL, *n* = 3) when compared with untreated mice (7.6 ± 0.1 nL, *n* = 3, *p* = 0.0047), although no difference was observed at 3 and 5 h (Supplementary Table S6). The volume of endolymph in noise-exposed mice treated with hypertonic saline was still significantly elevated compared with unexposed control mice at the 5 h (5.6 ± 0.1 nL, *n* = 3 vs. 4.7 ± 0.04 nL, *n* = 3, *p* = 0.01) and 7 h (6.0 ± 0.1 nL, *n* = 3 vs. 4.6 ± 0.1 nL, *n* = 3, *p* = 0.0057) time points. Endolymph volume was not significantly different at any time point following 100 dB SPL noise exposure between untreated mice and those treated with normotonic saline.

### Round Window Application of Hypertonic Saline in Noise-Exposed Ears Reduces Inner Hair Cell Synapse Loss in the Cochlear Base

Given that hypertonic saline ameliorates endolymphatic hydrops, we next sought to determine if it has any effect on noise-induced cochlear synaptopathy. We applied this solution to the left ears of mice through an intratympanic injection immediately following 100 dB SPL noise exposure. The untreated right ear served as one control, left ears treated with normotonic saline after noise exposure comprised another control group, and a final set of controls consisted of mice that were not exposed to noise. We counted the number of synaptic ribbons and PSDs per IHC 7 days after noise exposure as before. We did not count OHC ribbons or PSDs since we already showed that this noise exposure protocol did not alter the number of OHC synapses.

**FIGURE 6:**
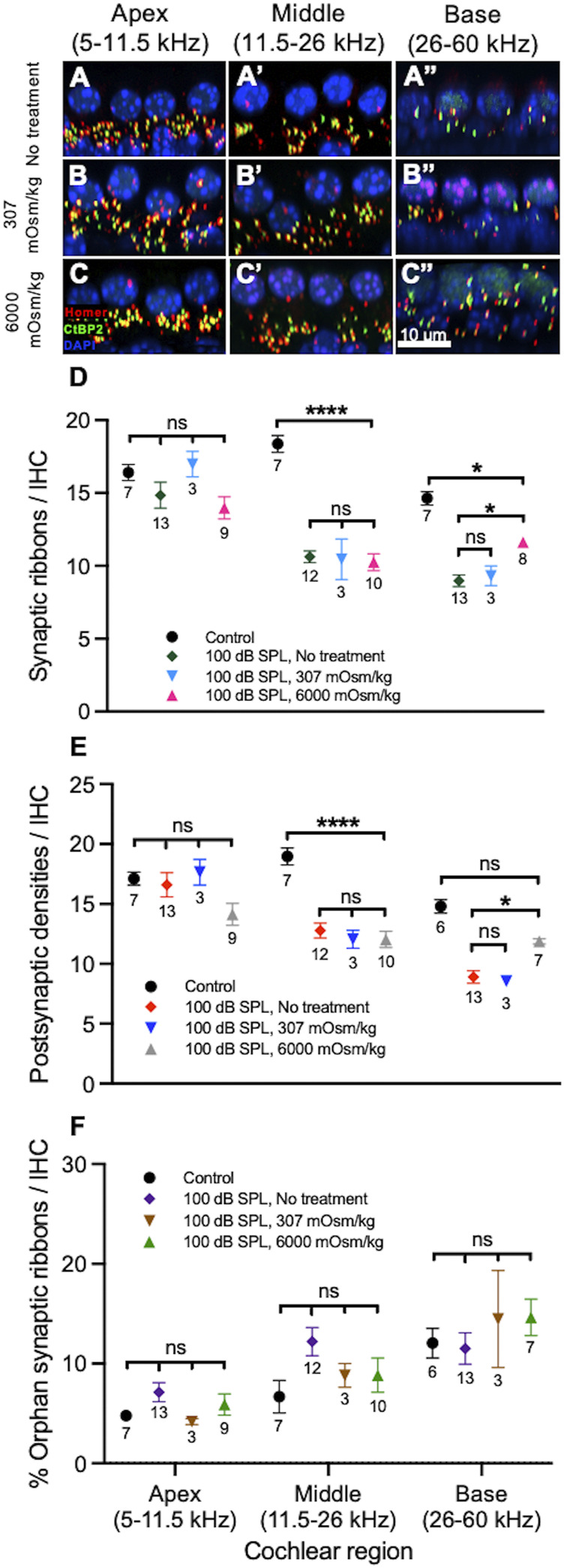
Osmotic treatment partially rescues synapse loss after 2 h of 100 decibel (dB) sound pressure level (SPL) noise exposure. (A–C) Representative sections from the organ of Corti of mice 7 days after 100 dB SPL noise exposure displaying 4 inner hair cell (IHC) nuclei and associated ribbons as well as postsynaptic densities (PSDs). Immunolabeling was performed to visualize IHC ribbons (CtBP2, green), PSDs (Homer, red) and nuclei (DAPI, blue). Right ears received no treatment **(A)**, while left ears received either normotonic saline (307 mOsm/kg) **(B)** or hypertonic saline (6,000 mOsm/kg) application to the middle ear after noise exposure **(C)**. More ribbons and PSDs per IHC are present in the cochlear base of ears treated with hypertonic saline (6,000 mOsm/kg) compared with ears treated with normotonic saline (307 mOsm/kg) and untreated ears (no treatment). **(D)** Quantification of ribbons per IHC. Mouse ears treated with hypertonic saline had increased numbers of ribbons per IHC when compared with untreated ears in the base of the cochlea. There were no significant differences between untreated ears and those treated with hypertonic saline in the apex or middle of the cochlea. There were also no significant differences between untreated ears and those treated with normotonic saline in any region of the cochlea. **(E)** Quantification of PSDs per IHC. Mouse ears treated with hypertonic saline had increased numbers of PSDs per IHC when compared with untreated ears in the base of the cochlea. There were no significant differences between untreated ears and those treated with hypertonic saline in the apex or middle of the cochlea. There were also no significant differences between untreated ears and those treated with normotonic saline in any region of the cochlea. **(F)** Comparison of the percentage of orphan ribbons (without an associated PSD) per IHC between different treatment groups. There were no significant differences in the percentage of orphan ribbons per IHC between mice treated with hypertonic saline, normotonic saline, untreated mice and control, unexposed mice in any region of the cochlea. Data points represent means and error bars indicate standard error. Sample sizes are displayed under each data point and expressed in number of mice. ns = not significant, *p < 0.05, ****p < 0.0001.

On visual inspection, it appeared that in the apical and middle regions of the cochlea there were no differences in the numbers of ribbons and PSDs per IHC between noise-exposed, untreated ears and those treated with normotonic or hypertonic saline (Figures 6A–C). More cochlear synapses per IHC were present in the cochlear base of noise-exposed ears treated with hypertonic saline compared with untreated ears and those treated with normotonic saline. The overall numbers of cochlear synapses were similar between noise-exposed and control ears in the apex but reduced in the middle and base of the cochlea in noise-exposed ears when compared with control ears.

Quantification of these structures was then performed as before (Figures 6D–F). Compared with control ears, there were significant reductions in the numbers of ribbons and PSDs per IHC in the middle of the cochlea among all groups exposed to 100 dB SPL noise (Supplementary Tables S7, 8). There were no significant differences in ribbons or PSDs per IHC in the middle of the cochlea when noise-exposed ears treated with hypertonic saline (10.3 ± 0.6 ribbons, *n* = 10 and 12.0 ± 0.7 PSDs, *n* = 10) were compared with untreated ears (10.6 ± 0.4 ribbons, *n* = 12 and 12.8 ± 0.6 PSDs, *n* = 12; *p* = 0.9692 for ribbons and *p* = 0.8661 for PSDs per IHC). There were also no significant differences in the numbers of ribbons and PSDs per IHC between untreated ears and those treated with normotonic saline (10.5 ± 1.4 ribbons, *n* = 3 and 12.1 ± 0.8 PSDs, *n* = 3; *p* = 0.9991 for ribbons and *p* = 0.9594 for PSDs per IHC) in the middle of the cochlea. In the base of the cochlea, there were significant reductions in the number of ribbons per IHC among all groups exposed to 100 dB SPL noise when compared with control ears. PSDs per IHC were only significantly reduced in untreated ears (8.9 ± 0.5, *n* = 13) and those treated with normotonic saline (8.5 ± 0.4, *n* = 3) when compared with controls (14.8 ± 0.6, *n* = 6; *p* < 0.0001 for comparison with untreated ears and *p* = 0.001 for comparison with ears treated with normotonic saline), while PSDs per IHC in ears treated with hypertonic saline after noise exposure (11.9 ± 0.2, *n* = 7) were not significantly different than controls (*p* = 0.1001). Most notably, noise-exposed ears treated with hypertonic saline had a significantly greater number of ribbons (11.6 ± 0.3, *n* = 8) and PSDs per IHC (11.9 ± 0.2, *n* = 7) in the base compared with untreated ears (9.0 ± 0.4 ribbons, *n* = 13 and 8.9 ± 0.5 PSDs, *n* = 13; *p* = 0.0132 for ribbons and *p* = 0.0315 for PSDs per IHC). Compared with controls, ears which were treated with hypertonic saline after exposure to 100 dB SPL nose had a 20.5 ± 3.5% reduction in ribbons and 19.8 ± 3.9% reduction in PSDs per IHC in the base of the cochlea. By comparison, ears that did not undergo treatment after 100 dB SPL noise exposure had a 38.7 ± 4.4% reduction in ribbons and 39.8 ± 5.8% reduction in PSDs per IHC compared with controls. There were no significant differences in the numbers of ribbons and PSDs between ears treated with normotonic saline and untreated ears in the base of the cochlea. In the apex, ribbon and PSD per IHC counts did not significantly differ between control ears and those exposed to 100 dB SPL, including untreated ears and those treated with hypertonic or normotonic saline. Additionally, the percentage of orphan ribbons per IHC did not significantly differ between any of the experimental groups in the apex, middle or base of the cochlea (Supplementary Table S9).

## Discussion

### Endolymphatic Hydrops and Inner Hair Cell Synaptopathy Occur at Similar Noise Intensity Thresholds

Herein, we demonstrate that a threshold level of traumatic noise exposure exists. Above this threshold, both endolymphatic hydrops and cochlear synaptopathy develop. Below this threshold, neither develops. This finding suggests that endolymphatic hydrops and cochlear synaptopathy may derive through a common mechanism. Furthermore, it argues that endolymphatic hydrops may develop via an “all-or-none” mechanism following prolonged noise exposure. One potential mechanism is that a large amount of noise-induced stereociliary damage may be necessary before the ability of stereociliary mechanoelectrical transduction (MET) channels to uptake potassium becomes less than the secretion of potassium into the endolymph by the stria vascularis (Wangemann, 2002; Zdebik et al., 2009; Salt and Plontke, 2010). Once this point is reached, potassium buildup occurs, leading to osmotic influx of water into the endolymph and the development of endolymphatic hydrops (Kim et al., 2018a).

In support of this mechanism, we have previously shown that Tecta^C1509G/C1509G^ mutant mice, in which the tectorial membrane is elevated off the cochlear epithelium, have increased endolymph volume compared with CBA/CaJ mice and do not develop excess endolymphatic hydrops in response to blast exposure (Xia et al., 2010; Kim et al., 2018a). We postulated that this is because the lack of static displacement of OHC stereociliary bundles by the tectorial membrane reduces potassium uptake through MET channels, increasing endolymph volume in these mice. After blast exposure, the tectorial membrane does not shear OHC stereocilia because it is detached from the organ of Corti, so endolymph volume does not increase further. It is possible that similar findings would also be observed in TMC1 mutant mice, or any other mouse mutant that has impaired MET channel currents (Kawashima et al., 2011; Fettiplace, 2016; Beurg et al., 2019).

Using our noise exposure protocol, we found that the threshold for the formation of endolymphatic hydrops, between 95–100 dB SPL, mirrors the threshold for noise-induced cochlear synaptopathy in CBA/CaJ mice. The sharp demarcation between synaptopathic and non-synaptopathic noise intensities is supported by a study from Jensen et al. (2015), which showed that 6-week old mice exposed to 2 h of 94 dB SPL noise developed a temporary threshold shift (TTS) without a corresponding loss of IHC ribbons, while those exposed to 97 dB SPL developed TTS and synaptopathy throughout the basal half of the cochlea. Hickox and Liberman (2014) similarly found that in 16–18 week old CBA/CaJ mice, a 100 dB SPL noise exposure reliably produced cochlear synaptopathy, whereas a 94 dB SPL stimulus did not, despite the fact that both produced a TTS of 40 dB SPL. While the reason for the sharp cutoff between synaptopathic and non-synaptopathic noise intensities is still unknown, our results argue that the development of endolymphatic hydrops plays a role, since both endolymphatic hydrops and cochlear synaptopathy have similar thresholds. Importantly, while our results demonstrate that endolymphatic hydrops is associated with cochlear synaptopathy following noise exposure, no conclusion about a causative relationship between endolymphatic hydrops and loss of synapses can be made based on the experiments performed in this study. Still, these results indicate that endolymphatic hydrops may be used as a surrogate marker for the loss of IHC ribbon synapses following prolonged noise exposure just as it does after blast trauma (Kim et al., 2018a).

### Endolymphatic Hydrops is not Correlated with Changes in the Number of Inner Hair Cell Orphan Ribbons or Outer Hair Cell Ribbons

Of note, we found no change in the percentage of IHC orphan ribbons 1 week after traumatic noise exposure. This finding is consistent with the results of prior studies, which have shown that while the number of orphans may increase and remain increased in number for at least 24 hours after noise trauma, most IHC ribbons are once again paired with postsynaptic elements by 1 week post-exposure in both CBA/CaJ (Liberman et al., 2015; Suzuki et al., 2016) and C57BL/6J mice (Kim et al., 2019a). The number of orphan ribbons in our study was larger than has been reported in prior studies on noise-exposed CBA/CaJ mice, which is likely due to the less robust labelling of Homer when compared with CtBP2 in our experiments (Liberman et al., 2015; Suzuki et al., 2016). Our results also indicated that there was no significant change in the number of ribbons per OHC 1 week after noise exposure, a finding supported by similar results observed by Zhao et al. (2021). Although we did not investigate the localization of ribbons relative to the nucleus of OHCs, a recent study noted an increase in ribbons at the OHC synaptic pole after traumatic noise exposure, despite finding no change in the total number of ribbons per OHC after noise (Wood et al., 2021). Thus, while our previous work showed that blast exposure results in the loss of OHC ribbons, it appears that cochlear trauma from noise exposure of approximately 100 dB SPL is not sufficient to cause a reduction in OHC ribbon numbers (Kim et al., 2018a; Wood et al., 2021; Zhao et al., 2021).

### Round Window Application of Hypertonic Saline Decreases Endolymphatic Hydrops and Partially Prevents the Loss of Inner Hair Cell Synapses After Traumatic Noise Exposure

Round window application of hypertonic saline decreased the degree of endolymphatic hydrops that developed following prolonged noise exposure when compared with untreated mice. The mechanism behind this effect is based on the principle of osmotic stabilization, which we have previously shown to be effective in reducing endolymphatic hydrops following blast exposure (Kim et al., 2018a). The hypertonic saline creates an osmotic gradient across the round window membrane, which drives water efflux from the perilymph. This efflux then creates a second osmotic gradient between perilymph and endolymph across RM, leading to water efflux from the scala media and a reduction in endolymph volume (Goycoolea, 2001; Duan and Zhi-qiang, 2009; Kim et al., 2018a). Importantly, endolymphatic hydrops still developed following round window application of hypertonic saline in our study, but to a lesser degree than in untreated mice. This may be because the rate of efflux driven by hypertonic saline and resorption of potassium by the damaged apical transduction channels is not sufficient to completely overcome the secretion of potassium by the stria vascularis and corresponding influx of water into the endolymph.

Nonetheless, we found that hypertonic saline treatment was able to partially rescue the loss of ribbons and PSDs in the base of the cochlea. This further supports the notion that endolymphatic hydrops may be a reliable surrogate marker for noise-induced synaptopathy since a reduction in endolymphatic hydrops was associated with a corresponding increase in cochlear synapses. The reason that hypertonic saline rescued synaptic loss in the base of the cochlea may be either through the reduction of endolymphatic hydrops, reduction of auditory dendrite terminal bouton swelling, or both. If endolymphatic hydrops contributes to synaptopathy by overstimulating IHCs and leading to glutamate excitotoxicity, then reducing the severity of endolymphatic hydrops would reduce the loss of ribbons (Kim et al., 2018a). Alternatively, the hypertonic solution may reduce postsynaptic terminal bouton swelling independent of its effect on endolymphatic hydrops. Glutamate excitotoxicity results in swelling of auditory nerve postsynaptic boutons by activating ligand-gated ion channels, causing a toxic entry of ions and water into the terminal bouton (Mayer and Westbrook, 1987; Choi and Rothman, 1990; Pujol and Puel, 1999; Kim et al., 2019a; Hu et al., 2020). Morphological studies have demonstrated that swelling, disorganization and damage of type I postsynaptic nerve terminals in the region of their synaptic contact with IHCs follows noise exposure, and this likely precedes synaptic breakdown and a corresponding loss of ribbons within IHCs (Spoendlin, 1971; Robertson, 1983; Puel et al., 1998). Therefore, the osmotic gradient established between postsynaptic boutons and the surrounding perilymph through round window application of hypertonic saline may be capable of reducing the toxic swelling of synaptic boutons following glutamate excitotoxicity, thus preventing destruction of the synapse and protecting IHC ribbons (Kim et al., 2018a). Therefore, endolymphatic hydrops and synaptopathy may occur by separate mechanisms, yet both appear to be activated at similar sound intensity thresholds between 95 and 100 dB SPL and affected by changes in perilymph osmolarity.

In the absence of noise exposure, the correlation between endolymphatic hydrops and synaptopathy is less clear. While we have previously shown that lowering perilymph osmolarity through round-window application of hypotonic saline causes the development of endolymphatic hydrops and a loss of synapses throughout the cochlea of CBA/CaJ mice (Kim et al., 2018a), Valenzuela et al. found that endolymphatic sac ablation in guinea pigs, which has been shown to cause histologically measurable endolymphatic hydrops by 30 postoperative days (Lee et al., 2020), did not result in a corresponding loss of cochlear synapses (Valenzuela et al., 2020). Unlike our prior study (Kim et al., 2018a), Valenzuela et al. did not visualize endolymphatic hydrops directly in live animals. Nonetheless, the lack of a clear relationship between endolymphatic hydrops and cochlear synaptopathy in the absence of noise exposure suggests that endolymphatic hydrops may only be a reliable surrogate marker for cochlear synaptopathy after noise trauma, although further research on this topic is needed.

### In the Cochlear Apex, Endolymphatic Hydrops and Inner Hair Cell Synaptopathy did not Correlate

Differences in IHC sensitivity to acoustic trauma or scala media distensibility throughout the cochlea may explain the discrepancy between the location of endolymphatic hydrops and the pattern of noise-induced cochlear synaptopathy identified in our study. Based on the orientation of the mouse cochlea and the angle of the OCT scanning laser, only the apical turn of the cochlea could be imaged, corresponding to the 9 kHz location on the cochlear tonotopic map. While endolymphatic hydrops was observed in the apex of the cochlea in response to 100 dB SPL noise exposure, loss of IHC synapses occurred throughout the middle and base of the cochlea and spared the apical region. This pattern of noise-induced synapse loss is well established and may be due to the increased sensitivity of IHCs in the basal half of the cochlea to acoustic trauma, possibly due to decreased levels of glutathione and increased susceptibility to reactive oxygen species (Sha et al., 2001; Hickox and Liberman, 2014; Liberman et al., 2015; Kim et al., 2019a). Alternatively, the discrepancy between the locations of endolymphatic hydrops and synaptopathy may be due to differences in the distensibility of the scala media between the apex, middle and base of the cochlea. The cochlear apex is the most distensible segment of the cochlea, partially driven by the reduced stiffness and widening of the basilar membrane in this segment (Kimura and Schuknecht, 1965; Lichtenhan et al., 2017). The greater distensibility of the apical sensory structures may make them less susceptible to pressure build-up from increased endolymph volume, thus protecting IHC synapses in this location from overstimulation, glutamate excitotoxicity and synaptopathy.

### Applications to Acoustic Trauma in Humans

That endolymphatic hydrops is a marker of synaptopathy following traumatic noise exposure could lead to potential novel techniques for detecting noise-induced cochlear synaptopathy in humans. In animals, cochlear synaptopathy can be diagnosed via the suprathreshold amplitude of wave 1 of the ABR (Bramhall et al., 2018). In humans, however, intersubject variability in ABR amplitude due to small signal-to-noise ratios and variability in head size, tissue conductivity, and electrode resistance limit the diagnostic utility of this technique (Nikiforidis et al., 1993; Liberman et al., 2016). The ratio of summating potential to action potential and the middle ear reflex have recently been suggested to be more reliable metrics for cochlear synaptopathy than ABR amplitudes in humans (Liberman et al., 2016; Wojtczak et al., 2017; Valero et al., 2018). Here, we showed that noise exposures with intensities sufficient to produce cochlear synaptopathy also result in endolymphatic hydrops which can be detected using OCT. Although the use of OCT for cochlear imaging is currently not performed in humans, an OCT device that images the cochlea through the ear canal may allow translation of this technology to humans (Monroy et al., 2017; Kim et al., 2018b; Kim et al., 2019b; Burwood et al., 2019; Lui et al., 2021).

A second application of our results is that hypertonic saline may be used to partially rescue IHC ribbon loss in the base of the cochlea following traumatic noise exposure. Intratympanic injections are relatively simple procedures that can be performed in the office, and round window delivery of medications is routinely performed for other otologic conditions, including the use of intratympanic steroids for sudden sensorineural hearing loss (Patel et al., 2019). While other treatment modalities have been suggested for the treatment of cochlear synaptopathy following acoustic trauma, including intratympanic application of neutrophin 3 for regeneration of cochlear synapses and inhibition of AMP-activated protein kinase, a mediator of cochlear synaptopathy, using siRNA-silencing techniques and administration of competitive inhibitors, these therapies are likely to require substantial time and research to prove their efficacy (Hill et al., 2016; Suzuki et al., 2016; Hu et al., 2020). An advantage of hypertonic saline is that it is commonly used in the nasal passageways to treat sinus disease. The middle ear, being an extension of the sinuses, is likely to be considered a safe space to apply hypertonic saline. Our results suggest that osmotic treatment could be investigated as a therapy for acute noise exposure, such as after being exposed to a gunshot, firecracker, or airbag deployment. It is important to note, however, that the absence of auditory metrics in our study should limit its interpretations to only the assessment of physical damage to the cochlea.

## Data Availability

The raw data supporting the conclusion of this article will be made available by the authors, without undue reservation.

## Supplementary Material

Supplementary Movie S1 Time-lapse series of optical coherence tomography cross-sectional images demonstrating no change in the position of Reissner’s membrane over time following exposure to 95 decibel sound pressure level noise. Time since the end of noise exposure is displayed in the bottom right corner.

Supplementary Movie S2 Time-lapse series of optical coherence tomography cross-sectional images demonstrating progressive bowing of Reissner’s membrane over time (endolymphatic hydrops) following exposure to 100 decibel sound pressure level noise. Time since the end of noise exposure is displayed in the bottom right corner.

Supplementary Figure S1 Cochleogram showing the average frequency ranges and percentages of total cochlear length from the base associated with the apical, middle and base segments, as well as the location where optical coherence tomography (OCT) was performed. Red dots indicate 10% intervals along the length of the cochlea, with 0% corresponding to the basal-most cochlear location. The base segment corresponds to the frequency range of 60 ± 8 − 26 ± 2 kHz (10.0% to 39.0% of total cochlear length), the middle segment corresponds to the frequency range of 26 ± 2 − 11.5 ± 0.5 kHz (39.0% to 69.3% of total cochlear length), and the apical segment corresponds to the frequency range of 11.5 ± 0.5 − 5 kHz (69.3% to 100.0% of total cochlear length). The hook region, corresponding to the frequency range of 80 − 60 ± 8 kHz (0.0 to 10.0% of total cochlear length), was not analyzed as it is easily injured during dissection. The 300-μm region where OCT was performed to image the cochlea, centered around the 9 kHz location, is marked (75.7%-80.3% of total cochlear length). Also shown is the 150-μm subsection of this region where endolymph volume of the scala media was measured.

Supplementary Figure S2 Automatic method for detecting, mapping, and counting ribbons and postsynaptic densities (PSDs). **(A)** Image of inner hair cells (IHCs) with CtBP2-labeled ribbons shown in green and Homer-labeled PSDs in red. Magnified image of a co-localized ribbon and PSD is shown in the bottom left corner. **(B)** From the CtBP2-labeled image, a 2D median filter of ±1.4 μm removes most of the smaller features of the image and just leaves the background. **(C)** A median filter of ±0.35 μm is used to smooth out the original data and reduce speckle noise. **(D)** Structures the size of ribbons are isolated by subtracting **(B)** from **(C)**. **(F)** Ribbons are identified by selecting only those structures with an intensity above a set threshold and with a height in the z-direction >0.48 μm (ribbons marked by blue circles). This same process is repeated for the Homer-labeled image to count PSDs.
