# Supplementary material for: Endolymphatic Hydrops is a Marker of Synaptopathy Following Traumatic Noise Exposure
Source: Front Cell Dev Biol. 2021 Nov 5;9:747870. doi: 10.3389/fcell.2021.747870 (PMC8602199; doi:10.3389/fcell.2021.747870)
Supplement: Supplementary file 2 [file Table1.DOCX]

Supplementary Table 1

| **Fig. 1E** |  |  |  |  |
| --- | --- | --- | --- | --- |
|  | W value | P value | Passed normality test (alpha=0.05)? |  |
| Shapiro-Wilk test for normality | 0.9614 | 0.1385 | Yes |  |
|  |  |  |  |  |
| Repeated measures two-way ANOVA | Sum of Squares | F value | P value | Significance |
| Interaction | 3.093 | 6.063 | 0.0005 | *** |
| Time | 0.4568 | 3.582 | 0.0789 | ns |
| Noise Intensity | 34.86 | 48.84 | <0.0001 | **** |
| Repeated Measures | 1.784 | 2.798 | 0.024 | * |
| Residual | 1.275 |  |  |  |
|  |  |  |  |  |
| Tukey's multiple comparisons test |  |  |  |  |
| 3 h | P value | Significance |  |  |
| Control (n=3) vs. 80 dB SPL (n=3) | >0.9999 | ns |  |  |
| Control (n=3) vs. 90 dB SPL (n=3) | 0.6874 | ns |  |  |
| Control (n=3) vs. 95 dB SPL (n=3) | 0.5285 | ns |  |  |
| Control (n=3) vs. 100 dB SPL (n=3) | 0.2386 | ns |  |  |
| 80 dB SPL (n=3) vs. 90 dB SPL (n=3) | 0.8435 | ns |  |  |
| 80 dB SPL (n=3) vs. 95 dB SPL (n=3) | 0.642 | ns |  |  |
| 80 dB SPL (n=3) vs. 100 dB SPL (n=3) | 0.2281 | ns |  |  |
| 90 dB SPL (n=3) vs. 95 dB SPL (n=3) | 0.106 | ns |  |  |
| 90 dB SPL (n=3) vs. 100 dB SPL (n=3) | 0.1902 | ns |  |  |
| 95 dB SPL (n=3) vs. 100 dB SPL (n=3) | 0.3228 | ns |  |  |
|  |  |  |  |  |
| 5 h |  |  |  |  |
| Control (n=3) vs. 80 dB SPL (n=3) | 0.3922 | ns |  |  |
| Control (n=3) vs. 90 dB SPL (n=3) | 0.3213 | ns |  |  |
| Control (n=3) vs. 95 dB SPL (n=3) | 0.4307 | ns |  |  |
| Control (n=3) vs. 100 dB SPL (n=3) | 0.0496 | * |  |  |
| 80 dB SPL (n=3) vs. 90 dB SPL (n=3) | >0.9999 | ns |  |  |
| 80 dB SPL (n=3) vs. 95 dB SPL (n=3) | 0.1337 | ns |  |  |
| 80 dB SPL (n=3) vs. 100 dB SPL (n=3) | 0.0291 | * |  |  |
| 90 dB SPL (n=3) vs. 95 dB SPL (n=3) | 0.113 | ns |  |  |
| 90 dB SPL (n=3) vs. 100 dB SPL (n=3) | 0.0327 | * |  |  |
| 95 dB SPL (n=3) vs. 100 dB SPL (n=3) | 0.0485 | * |  |  |
|  |  |  |  |  |
| 7 h |  |  |  |  |
| Control (n=3) vs. 80 dB SPL (n=3) | 0.9353 | ns |  |  |
| Control (n=3) vs. 90 dB SPL (n=3) | >0.9999 | ns |  |  |
| Control (n=3) vs. 95 dB SPL (n=3) | 0.9277 | ns |  |  |
| Control (n=3) vs. 100 dB SPL (n=3) | 0.0006 | *** |  |  |
| 80 dB SPL (n=3) vs. 90 dB SPL (n=3) | 0.9467 | ns |  |  |
| 80 dB SPL (n=3) vs. 95 dB SPL (n=3) | 0.7433 | ns |  |  |
| 80 dB SPL (n=3) vs. 100 dB SPL (n=3) | 0.0006 | *** |  |  |
| 90 dB SPL (n=3) vs. 95 dB SPL (n=3) | 0.9222 | ns |  |  |
| 90 dB SPL (n=3) vs. 100 dB SPL (n=3) | 0.0006 | *** |  |  |
| 95 dB SPL (n=3) vs. 100 dB SPL (n=3) | 0.0121 | * |  |  |

ns = not significant, *P<0.05, ***P<0.001, ****P<0.0001.
