# Supplementary material for: Endolymphatic Hydrops is a Marker of Synaptopathy Following Traumatic Noise Exposure
Source: Front Cell Dev Biol. 2021 Nov 5;9:747870. doi: 10.3389/fcell.2021.747870 (PMC8602199; doi:10.3389/fcell.2021.747870)
Supplement: Supplementary file 3 [file Table8.DOCX]

Supplementary Table 8

| **Fig. 6E** |  |  |  |  |
| --- | --- | --- | --- | --- |
|  | W value | P value | Passed normality test (alpha=0.05)? |  |
| Shapiro-Wilk test for normality | 0.9904 | 0.7415 | Yes | ns |
|  |  |  |  |  |
| Two-way ANOVA | Sum of Squares (Type III) | F value | P value | Significance |
| Interaction | 171.5 | 5.613 | <0.0001 | **** |
| Cochlear Region | 330.7 | 32.47 | <0.0001 | **** |
| Treatment Type | 278.6 | 18.24 | <0.0001 | **** |
| Residual | 412.4 |  |  |  |
|  |  |  |  |  |
| Tukey's multiple comparisons test |  |  |  |  |
| Apex (5-11.5 kHz) | P value | Significance |  |  |
| Control (n=7) vs. 100 dB SPL, No treatment (n=13) | 0.9623 | ns |  |  |
| Control (n=7) vs. 100 dB SPL, 6000 mOsm/kg (n=9) | 0.0508 | ns |  |  |
| Control (n=7) vs. 100 dB SPL, 307 mOsm/kg (n=3) | 0.9863 | ns |  |  |
| 100 dB SPL, No treatment (n=13) vs. 100 dB SPL, 6000 mOsm/kg (n=9) | 0.0647 | ns |  |  |
| 100 dB SPL, No treatment (n=13) vs. 100 dB SPL, 307 mOsm/kg (n=3) | 0.8882 | ns |  |  |
| 100 dB SPL, 6000 mOsm/kg (n=9) vs. 100 dB SPL, 307 mOsm/kg (n=3) | 0.0995 | ns |  |  |
|  |  |  |  |  |
| Middle (11.5-26 kHz) |  |  |  |  |
| Control (n=7) vs. 100 dB SPL, No treatment (n=12) | <0.0001 | **** |  |  |
| Control (n=7) vs. 100 dB SPL, 6000 mOsm/kg (n=10) | <0.0001 | **** |  |  |
| Control (n=7) vs. 100 dB SPL, 307 mOsm/kg (n=3) | 0.0002 | *** |  |  |
| 100 dB SPL, No treatment (n=12) vs. 100 dB SPL, 6000 mOsm/kg (n=10) | 0.8661 | ns |  |  |
| 100 dB SPL, No treatment (n=12) vs. 100 dB SPL, 307 mOsm/kg (n=3) | 0.9594 | ns |  |  |
| 100 dB SPL, 6000 mOsm/kg (n=10) vs. 100 dB SPL, 307 mOsm/kg (n=3) | >0.9999 | ns |  |  |
|  |  |  |  |  |
| Base (26-60 kHz) |  |  |  |  |
| Control (n=6) vs. 100 dB SPL, No treatment (n=13) | <0.0001 | **** |  |  |
| Control (n=6) vs. 100 dB SPL, 6000 mOsm/kg (n=7) | 0.1001 | ns |  |  |
| Control (n=6) vs. 100 dB SPL, 307 mOsm/kg (n=3) | 0.0010 | ** |  |  |
| 100 dB SPL, No treatment (n=13) vs. 100 dB SPL, 6000 mOsm/kg (n=7) | 0.0315 | * |  |  |
| 100 dB SPL, No treatment (n=13) vs. 100 dB SPL, 307 mOsm/kg (n=3) | 0.9942 | ns |  |  |
| 100 dB SPL, 6000 mOsm/kg (n=7) vs. 100 dB SPL, 307 mOsm/kg (n=3) | 0.1493 | ns |  |  |

ns = not significant, *P<0.05, **P<0.01, ***P<0.001, ****P<0.0001.
