# Supplementary material for: Endolymphatic Hydrops is a Marker of Synaptopathy Following Traumatic Noise Exposure
Source: Front Cell Dev Biol. 2021 Nov 5;9:747870. doi: 10.3389/fcell.2021.747870 (PMC8602199; doi:10.3389/fcell.2021.747870)
Supplement: Supplementary file 5 [file Table7.DOCX]

Supplementary Table 7

| **Fig. 6D** |  |  |  |  |
| --- | --- | --- | --- | --- |
|  | W value | P value | Passed normality test (alpha=0.05)? |  |
| Shapiro-Wilk test for normality | 0.9878 | 0.531 | Yes |  |
|  |  |  |  |  |
| Two-way ANOVA | Sum of Squares (Type III) | F value | P value | Significance |
| Interaction | 160.7 | 7.434 | <0.0001 | **** |
| Cochlear Region | 246.7 | 34.23 | <0.0001 | **** |
| Treatment Type | 370.8 | 34.31 | <0.0001 | **** |
| Residual | 299.1 |  |  |  |
|  |  |  |  |  |
| Tukey's multiple comparisons test |  |  |  |  |
| Apex (5-11.5 kHz) | P value | Significance |  |  |
| Control (n=7) vs. 100 dB SPL, No treatment (n=13) | 0.3039 | ns |  |  |
| Control (n=7) vs. 100 dB SPL, 6000 mOsm/kg (n=9) | 0.0614 | ns |  |  |
| Control (n=7) vs. 100 dB SPL, 307 mOsm/kg (n=3) | 0.9716 | ns |  |  |
| 100 dB SPL, No treatment (n=13) vs. 100 dB SPL, 6000 mOsm/kg (n=9) | 0.7173 | ns |  |  |
| 100 dB SPL, No treatment (n=13) vs. 100 dB SPL, 307 mOsm/kg (n=3) | 0.3026 | ns |  |  |
| 100 dB SPL, 6000 mOsm/kg (n=9) vs. 100 dB SPL, 307 mOsm/kg (n=3) | 0.0905 | ns |  |  |
|  |  |  |  |  |
| Middle (11.5-26 kHz) |  |  |  |  |
| Control (n=7) vs. 100 dB SPL, No treatment (n=12) | <0.0001 | **** |  |  |
| Control (n=7) vs. 100 dB SPL, 6000 mOsm/kg (n=10) | <0.0001 | **** |  |  |
| Control (n=7) vs. 100 dB SPL, 307 mOsm/kg (n=3) | <0.0001 | **** |  |  |
| 100 dB SPL, No treatment (n=12) vs. 100 dB SPL, 6000 mOsm/kg (n=10) | 0.9692 | ns |  |  |
| 100 dB SPL, No treatment (n=12) vs. 100 dB SPL, 307 mOsm/kg (n=3) | 0.9991 | ns |  |  |
| 100 dB SPL, 6000 mOsm/kg (n=10) vs. 100 dB SPL, 307 mOsm/kg (n=3) | 0.9985 | ns |  |  |
|  |  |  |  |  |
| Base (26-60 kHz) |  |  |  |  |
| Control (n=7) vs. 100 dB SPL, No treatment (n=13) | <0.0001 | **** |  |  |
| Control (n=7) vs. 100 dB SPL, 6000 mOsm/kg (n=8) | 0.0156 | * |  |  |
| Control (n=7) vs. 100 dB SPL, 307 mOsm/kg (n=3) | 0.0006 | *** |  |  |
| 100 dB SPL, No treatment (n=13) vs. 100 dB SPL, 6000 mOsm/kg (n=8) | 0.0132 | * |  |  |
| 100 dB SPL, No treatment (n=13) vs. 100 dB SPL, 307 mOsm/kg (n=3) | 0.9921 | ns |  |  |
| 100 dB SPL, 6000 mOsm/kg (n=8) vs. 100 dB SPL, 307 mOsm/kg (n=3) | 0.2799 | ns |  |  |

ns = not significant, *P<0.05, ***P<0.001, ****P<0.0001.
