# Supplementary material for: Endolymphatic Hydrops is a Marker of Synaptopathy Following Traumatic Noise Exposure
Source: Front Cell Dev Biol. 2021 Nov 5;9:747870. doi: 10.3389/fcell.2021.747870 (PMC8602199; doi:10.3389/fcell.2021.747870)
Supplement: Supplementary file 7 [file Table9.DOCX]

Supplementary Table 9

| **Fig. 6F** |  |  |  |  |
| --- | --- | --- | --- | --- |
|  | W value | P value | Passed normality test (alpha=0.05)? |  |
| Shapiro-Wilk test for normality | 0.9795 | 0.1514 | Yes |  |
|  |  |  |  |  |
| Two-way ANOVA | Sum of Squares (Type III) | F value | P value | Significance |
| Interaction | 161.1 | 1.35 | 0.2448 | ns |
| Cochlear Region | 679.6 | 17.09 | <0.0001 | **** |
| Treatment Type | 81.37 | 1.364 | 0.2598 | ns |
| Residual | 1611 |  |  |  |
|  |  |  |  |  |
| Tukey's multiple comparisons test |  |  |  |  |
| Apex (5-11.5 kHz) | P value | Significance |  |  |
| Control (n=7) vs. 100 dB SPL, No treatment (n=13) | 0.6749 | ns |  |  |
| Control (n=7) vs. 100 dB SPL, 6000 mOsm/kg (n=9) | 0.9587 | ns |  |  |
| Control (n=7) vs. 100 dB SPL, 307 mOsm/kg (n=3) | 0.9974 | ns |  |  |
| 100 dB SPL, No treatment (n=13) vs. 100 dB SPL, 6000 mOsm/kg (n=9) | 0.9203 | ns |  |  |
| 100 dB SPL, No treatment (n=13) vs. 100 dB SPL, 307 mOsm/kg (n=3) | 0.7308 | ns |  |  |
| 100 dB SPL, 6000 mOsm/kg (n=9) vs. 100 dB SPL, 307 mOsm/kg (n=3) | 0.9381 | ns |  |  |
|  |  |  |  |  |
| Middle (11.5-26 kHz) |  |  |  |  |
| Control (n=7) vs. 100 dB SPL, No treatment (n=12) | 0.0529 | ns |  |  |
| Control (n=7) vs. 100 dB SPL, 6000 mOsm/kg (n=10) | 0.7605 | ns |  |  |
| Control (n=7) vs. 100 dB SPL, 307 mOsm/kg (n=3) | 0.899 | ns |  |  |
| 100 dB SPL, No treatment (n=12) vs. 100 dB SPL, 6000 mOsm/kg (n=10) | 0.3007 | ns |  |  |
| 100 dB SPL, No treatment (n=12) vs. 100 dB SPL, 307 mOsm/kg (n=3) | 0.6452 | ns |  |  |
| 100 dB SPL, 6000 mOsm/kg (n=10) vs. 100 dB SPL, 307 mOsm/kg (n=3) | >0.9999 | ns |  |  |
|  |  |  |  |  |
| Base (26-60 kHz) |  |  |  |  |
| Control (n=6) vs. 100 dB SPL, No treatment (n=13) | 0.9949 | ns |  |  |
| Control (n=6) vs. 100 dB SPL, 6000 mOsm/kg (n=7) | 0.7273 | ns |  |  |
| Control (n=6) vs. 100 dB SPL, 307 mOsm/kg (n=3) | 0.8703 | ns |  |  |
| 100 dB SPL, No treatment (n=13) vs. 100 dB SPL, 6000 mOsm/kg (n=7) | 0.4485 | ns |  |  |
| 100 dB SPL, No treatment (n=13) vs. 100 dB SPL, 307 mOsm/kg (n=3) | 0.7314 | ns |  |  |
| 100 dB SPL, 6000 mOsm/kg (n=7) vs. 100 dB SPL, 307 mOsm/kg (n=3) | >0.9999 | ns |  |  |

ns = not significant, ****P<0.0001.
