# Supplementary material for: Endolymphatic Hydrops is a Marker of Synaptopathy Following Traumatic Noise Exposure
Source: Front Cell Dev Biol. 2021 Nov 5;9:747870. doi: 10.3389/fcell.2021.747870 (PMC8602199; doi:10.3389/fcell.2021.747870)
Supplement: Supplementary file 8 [file Table2.DOCX]

Supplementary Table 2

| **Fig. 2D** |  |  |  |  |
| --- | --- | --- | --- | --- |
|  | W value | P value | Passed normality test (alpha=0.05)? |  |
| Shapiro-Wilk Test for normality | 0.9877 | 0.6885 | Yes |  |
|  |  |  |  |  |
| Two-way ANOVA | Sum of Squares (Type III) | F value | P value | Significance |
| Interaction | 57.97 | 1.959 | 0.0674 | ns |
| Cochlear Region | 93.19 | 12.60 | <0.0001 | **** |
| Noise Intensity | 299.2 | 20.22 | <0.0001 | **** |
| Residual | 221.9 |  |  |  |
|  |  |  |  |  |
| Tukey's multiple comparisons test |  |  |  |  |
|  |  |  |  |  |
| Apex (5-11.5 kHz) | P value | Significance |  |  |
| Control (n=7) vs. 80 dB SPL (n=4) | 0.9492 | ns |  |  |
| Control (n=7) vs. 90 dB SPL (n=5) | 0.9756 | ns |  |  |
| Control (n=7) vs. 95 dB SPL (n=4) | 0.9619 | ns |  |  |
| Control (n=7) vs. 100 dB SPL (n=5) | 0.1001 | ns |  |  |
| 80 dB SPL (n=4) vs. 90 dB SPL (n=5) | 0.7526 | ns |  |  |
| 80 dB SPL (n=4) vs. 95 dB SPL (n=4) | >0.9999 | ns |  |  |
| 80 dB SPL (n=4) vs. 100 dB SPL (n=5) | 0.5551 | ns |  |  |
| 90 dB SPL (n=5) vs. 95 dB SPL (n=4) | 0.7821 | ns |  |  |
| 90 dB SPL (n=5) vs. 100 dB SPL (n=5) | 0.0421 | * |  |  |
| 95 dB SPL (n=4) vs. 100 dB SPL (n=5) | 0.5215 | ns |  |  |
|  |  |  |  |  |
| Middle (11.5-26 kHz) |  |  |  |  |
| Control (n=7) vs. 80 dB SPL (n=4) | 0.7958 | ns |  |  |
| Control (n=7) vs. 90 dB SPL (n=5) | 0.9575 | ns |  |  |
| Control (n=7) vs. 95 dB SPL (n=4) | 0.402 | ns |  |  |
| Control (n=7) vs. 100 dB SPL (n=5) | <0.0001 | **** |  |  |
| 80 dB SPL (n=4) vs. 90 dB SPL (n=5) | 0.4725 | ns |  |  |
| 80 dB SPL (n=4) vs. 95 dB SPL (n=4) | 0.9783 | ns |  |  |
| 80 dB SPL (n=4) vs. 100 dB SPL (n=5) | 0.0016 | ** |  |  |
| 90 dB SPL (n=5) vs. 95 dB SPL (n=4) | 0.1741 | ns |  |  |
| 90 dB SPL (n=5) vs. 100 dB SPL (n=5) | <0.0001 | **** |  |  |
| 95 dB SPL (n=4) vs. 100 dB SPL (n=5) | 0.0105 | * |  |  |
|  |  |  |  |  |
| Base (26-60 kHz) |  |  |  |  |
| Control (n=7) vs. 80 dB SPL (n=4) | 0.3307 | ns |  |  |
| Control (n=7) vs. 90 dB SPL (n=5) | >0.9999 | ns |  |  |
| Control (n=7) vs. 95 dB SPL (n=4) | 0.8872 | ns |  |  |
| Control (n=7) vs. 100 dB SPL (n=5) | <0.0001 | **** |  |  |
| 80 dB SPL (n=4) vs. 90 dB SPL (n=5) | 0.4307 | ns |  |  |
| 80 dB SPL (n=4) vs. 95 dB SPL (n=4) | 0.1047 | ns |  |  |
| 80 dB SPL (n=4) vs. 100 dB SPL (n=5) | <0.0001 | **** |  |  |
| 90 dB SPL (n=5) vs. 95 dB SPL (n=4) | 0.8901 | ns |  |  |
| 90 dB SPL (n=5) vs. 100 dB SPL (n=5) | 0.0003 | *** |  |  |
| 95 dB SPL (n=4) vs. 100 dB SPL (n=5) | 0.0118 | * |  |  |

ns = not significant, *P<0.05, **P<0.01, ***P<0.001, ****P<0.0001.
