# Supplementary material for: Endolymphatic Hydrops is a Marker of Synaptopathy Following Traumatic Noise Exposure
Source: Front Cell Dev Biol. 2021 Nov 5;9:747870. doi: 10.3389/fcell.2021.747870 (PMC8602199; doi:10.3389/fcell.2021.747870)
Supplement: Supplementary file 9 [file Table5.DOCX]

Supplementary Table 5

| **Fig. 4D** |  |  |  |  |
| --- | --- | --- | --- | --- |
|  | W value | P value | Passed normality test (alpha=0.05)? |  |
| Shapiro-Wilk Test for normality | 0.9932 | 0.9636 | Yes |  |
|  |  |  |  |  |
| Two-way ANOVA | Sum of Squares (Type III) | F value | P value | Significance |
| Interaction | 0.7504 | 2.195 | 0.0403 | * |
| Cochlear Region | 5.654 | 66.16 | <0.0001 | **** |
| Noise Intensity | 0.07911 | 0.4629 | 0.7627 | ns |
| Residual | 2.564 |  |  |  |
|  |  |  |  |  |
| Tukey's multiple comparisons test |  |  |  |  |
| Apex (5-11.5 kHz) | P value | Significance |  |  |
| Control (n=7) vs. 80 dB SPL (n=4) | 0.6687 | ns |  |  |
| Control (n=7) vs. 90 dB SPL (n=5) | 0.673 | ns |  |  |
| Control (n=7) vs. 95 dB SPL (n=4) | 0.2905 | ns |  |  |
| Control (n=7) vs. 100 dB SPL (n=5) | 0.1348 | ns |  |  |
| 80 dB SPL (n=4) vs. 90 dB SPL (n=5) | >0.9999 | ns |  |  |
| 80 dB SPL (n=4) vs. 95 dB SPL (n=4) | 0.9797 | ns |  |  |
| 80 dB SPL (n=4) vs. 100 dB SPL (n=5) | 0.9211 | ns |  |  |
| 90 dB SPL (n=5) vs. 95 dB SPL (n=4) | 0.9593 | ns |  |  |
| 90 dB SPL (n=5) vs. 100 dB SPL (n=5) | 0.8669 | ns |  |  |
| 95 dB SPL (n=4) vs. 100 dB SPL (n=5) | 0.9993 | ns |  |  |
|  |  |  |  |  |
| Middle (11.5-26 kHz) |  |  |  |  |
| Control (n=7) vs. 80 dB SPL (n=4) | >0.9999 | ns |  |  |
| Control (n=7) vs. 90 dB SPL (n=5) | 0.5815 | ns |  |  |
| Control (n=7) vs. 95 dB SPL (n=4) | 0.7447 | ns |  |  |
| Control (n=7) vs. 100 dB SPL (n=5) | 0.4178 | ns |  |  |
| 80 dB SPL (n=4) vs. 90 dB SPL (n=5) | 0.6912 | ns |  |  |
| 80 dB SPL (n=4) vs. 95 dB SPL (n=4) | 0.813 | ns |  |  |
| 80 dB SPL (n=4) vs. 100 dB SPL (n=5) | 0.5463 | ns |  |  |
| 90 dB SPL (n=5) vs. 95 dB SPL (n=4) | 0.9999 | ns |  |  |
| 90 dB SPL (n=5) vs. 100 dB SPL (n=5) | 0.9992 | ns |  |  |
| 95 dB SPL (n=4) vs. 100 dB SPL (n=5) | 0.9951 | ns |  |  |
|  |  |  |  |  |
| Base (26-60 kHz) |  |  |  |  |
| Control (n=7) vs. 80 dB SPL (n=4) | 0.9967 | ns |  |  |
| Control (n=7) vs. 90 dB SPL (n=5) | 0.8121 | ns |  |  |
| Control (n=7) vs. 95 dB SPL (n=4) | 0.7991 | ns |  |  |
| Control (n=7) vs. 100 dB SPL (n=5) | 0.0841 | ns |  |  |
| 80 dB SPL (n=4) vs. 90 dB SPL (n=5) | 0.971 | ns |  |  |
| 80 dB SPL (n=4) vs. 95 dB SPL (n=4) | 0.9609 | ns |  |  |
| 80 dB SPL (n=4) vs. 100 dB SPL (n=5) | 0.3094 | ns |  |  |
| 90 dB SPL (n=5) vs. 95 dB SPL (n=4) | >0.9999 | ns |  |  |
| 90 dB SPL (n=5) vs. 100 dB SPL (n=5) | 0.6331 | ns |  |  |
| 95 dB SPL (n=4) vs. 100 dB SPL (n=5) | 0.7369 | ns |  |  |

ns = not significant, *P<0.05, ****P<0.0001.
