# Supplementary material for: Endolymphatic Hydrops is a Marker of Synaptopathy Following Traumatic Noise Exposure
Source: Front Cell Dev Biol. 2021 Nov 5;9:747870. doi: 10.3389/fcell.2021.747870 (PMC8602199; doi:10.3389/fcell.2021.747870)
Supplement: Supplementary file 10 [file Table3.DOCX]

Supplementary Table 3

| **Fig. 2E** |  |  |  |  |
| --- | --- | --- | --- | --- |
|  | W value | P value | Passed normality test (alpha=0.05)? |  |
| Shapiro-Wilk test for normality | 0.9787 | 0.2460 | Yes |  |
|  |  |  |  |  |
| Two-way ANOVA | Sum of Squares (Type III) | F value | P value | Significance |
| Interaction | 50.97 | 1.509 | 0.1738 | ns |
| Cochlear Region | 326.1 | 38.62 | <0.0001 | **** |
| Noise Intensity | 228.1 | 13.5 | <0.0001 | **** |
| Residual | 249.1 |  |  |  |
|  |  |  |  |  |
| Tukey's multiple comparisons test |  |  |  |  |
| Apex (5-11.5 kHz) | P value | Significance |  |  |
| Control (n=7) vs. 80 dB SPL (n=4) | 0.9998 | ns |  |  |
| Control (n=7) vs. 90 dB SPL (n=5) | 0.6223 | ns |  |  |
| Control (n=7) vs. 95 dB SPL (n=4) | 0.9701 | ns |  |  |
| Control (n=7) vs. 100 dB SPL (n=5) | 0.6458 | ns |  |  |
| 80 dB SPL (n=4) vs. 90 dB SPL (n=5) | 0.6293 | ns |  |  |
| 80 dB SPL (n=4) vs. 95 dB SPL (n=4) | 0.9947 | ns |  |  |
| 80 dB SPL (n=4) vs. 100 dB SPL (n=5) | 0.8396 | ns |  |  |
| 90 dB SPL (n=5) vs. 95 dB SPL (n=4) | 0.3728 | ns |  |  |
| 90 dB SPL (n=5) vs. 100 dB SPL (n=5) | 0.0872 | ns |  |  |
| 95 dB SPL (n=4) vs. 100 dB SPL (n=5) | 0.9722 | ns |  |  |
|  |  |  |  |  |
| Middle (11.5-26 kHz) |  |  |  |  |
| Control (n=7) vs. 80 dB SPL (n=4) | 0.8627 | ns |  |  |
| Control (n=7) vs. 90 dB SPL (n=5) | 0.936 | ns |  |  |
| Control (n=7) vs. 95 dB SPL (n=4) | 0.8675 | ns |  |  |
| Control (n=7) vs. 100 dB SPL (n=5) | <0.0001 | **** |  |  |
| 80 dB SPL (n=4) vs. 90 dB SPL (n=5) | 0.506 | ns |  |  |
| 80 dB SPL (n=4) vs. 95 dB SPL (n=4) | >0.9999 | ns |  |  |
| 80 dB SPL (n=4) vs. 100 dB SPL (n=5) | 0.0079 | ** |  |  |
| 90 dB SPL (n=5) vs. 95 dB SPL (n=4) | 0.5125 | ns |  |  |
| 90 dB SPL (n=5) vs. 100 dB SPL (n=5) | <0.0001 | **** |  |  |
| 95 dB SPL (n=4) vs. 100 dB SPL (n=5) | 0.0077 | ** |  |  |
|  |  |  |  |  |
| Base (26-60 kHz) |  |  |  |  |
| Control (n=6) vs. 80 dB SPL (n=4) | 0.3648 | ns |  |  |
| Control (n=6) vs. 90 dB SPL (n=5) | 0.7872 | ns |  |  |
| Control (n=6) vs. 95 dB SPL (n=4) | 0.8866 | ns |  |  |
| Control (n=6) vs. 100 dB SPL (n=5) | 0.0002 | *** |  |  |
| 80 dB SPL (n=4) vs. 90 dB SPL (n=5) | 0.9464 | ns |  |  |
| 80 dB SPL (n=4) vs. 95 dB SPL (n=4) | 0.9202 | ns |  |  |
| 80 dB SPL (n=4) vs. 100 dB SPL (n=5) | 0.1412 | ns |  |  |
| 90 dB SPL (n=5) vs. 95 dB SPL (n=4) | >0.9999 | ns |  |  |
| 90 dB SPL (n=5) vs. 100 dB SPL (n=5) | 0.0142 | * |  |  |
| 95 dB SPL (n=4) vs. 100 dB SPL (n=5) | 0.0165 | * |  |  |

ns = not significant, *P<0.05, **P<0.01, ***P<0.001, ****P<0.0001.
