# Supplementary material for: Endolymphatic Hydrops is a Marker of Synaptopathy Following Traumatic Noise Exposure
Source: Front Cell Dev Biol. 2021 Nov 5;9:747870. doi: 10.3389/fcell.2021.747870 (PMC8602199; doi:10.3389/fcell.2021.747870)
Supplement: Supplementary file 11 [file Table4.DOCX]

Supplementary Table 4

| **Fig. 2F** |  |  |  |  |
| --- | --- | --- | --- | --- |
|  | W value | P value | Passed normality test (alpha=0.05)? |  |
| Shapiro-Wilk (W) Test for Normality | 0.9694 | 0.0691 | Yes |  |
|  |  |  |  |  |
| Two-way ANOVA | Sum of Squares (Type III) | F value | P value | Significance |
| Interaction | 135.5 | 0.9322 | 0.4971 | ns |
| Cochlear Region | 251.1 | 6.909 | 0.002 | ** |
| Noise Intensity | 101.8 | 1.4 | 0.245 | ns |
| Residual | 1072 |  |  |  |
|  |  |  |  |  |
| Tukey's multiple comparisons test |  |  |  |  |
| Apex (5-11.5 kHz) | P Value | Significance |  |  |
| Control (n=7) vs. 80 dB SPL (n=4) | 0.8833 | ns |  |  |
| Control (n=7) vs. 90 dB SPL (n=5) | 0.9665 | ns |  |  |
| Control (n=7) vs. 95 dB SPL (n=4) | 0.9879 | ns |  |  |
| Control (n=7) vs. 100 dB SPL (n=5) | 0.5243 | ns |  |  |
| 80 dB SPL (n=4) vs. 90 dB SPL (n=5) | 0.998 | ns |  |  |
| 80 dB SPL (n=4) vs. 95 dB SPL (n=4) | 0.9949 | ns |  |  |
| 80 dB SPL (n=4) vs. 100 dB SPL (n=5) | 0.9874 | ns |  |  |
| 90 dB SPL (n=5) vs. 95 dB SPL (n=4) | >0.9999 | ns |  |  |
| 90 dB SPL (n=5) vs. 100 dB SPL (n=5) | 0.9132 | ns |  |  |
| 95 dB SPL (n=4) vs. 100 dB SPL (n=5) | 0.8923 | ns |  |  |
|  |  |  |  |  |
| Middle (11.5-26 kHz) |  |  |  |  |
| Control (n=7) vs. 80 dB SPL (n=4) | >0.9999 | ns |  |  |
| Control (n=7) vs. 90 dB SPL (n=5) | 0.8864 | ns |  |  |
| Control (n=7) vs. 95 dB SPL (n=4) | 0.957 | ns |  |  |
| Control (n=7) vs. 100 dB SPL (n=5) | 0.2767 | ns |  |  |
| 80 dB SPL (n=4) vs. 90 dB SPL (n=5) | 0.9024 | ns |  |  |
| 80 dB SPL (n=4) vs. 95 dB SPL (n=4) | 0.9823 | ns |  |  |
| 80 dB SPL (n=4) vs. 100 dB SPL (n=5) | 0.4574 | ns |  |  |
| 90 dB SPL (n=5) vs. 95 dB SPL (n=4) | 0.595 | ns |  |  |
| 90 dB SPL (n=5) vs. 100 dB SPL (n=5) | 0.0642 | ns |  |  |
| 95 dB SPL (n=4) vs. 100 dB SPL (n=5) | 0.8054 | ns |  |  |
|  |  |  |  |  |
| Base (26-60 kHz) |  |  |  |  |
| Control (n=6) vs. 80 dB SPL (n=4) | 0.973 | ns |  |  |
| Control (n=6) vs. 90 dB SPL (n=5) | >0.9999 | ns |  |  |
| Control (n=6) vs. 95 dB SPL (n=4) | 0.7049 | ns |  |  |
| Control (n=6) vs. 100 dB SPL (n=5) | >0.9999 | ns |  |  |
| 80 dB SPL (n=4) vs. 90 dB SPL (n=5) | 0.9559 | ns |  |  |
| 80 dB SPL (n=4) vs. 95 dB SPL (n=4) | 0.9729 | ns |  |  |
| 80 dB SPL (n=4) vs. 100 dB SPL (n=5) | 0.9851 | ns |  |  |
| 90 dB SPL (n=5) vs. 95 dB SPL (n=4) | 0.6659 | ns |  |  |
| 90 dB SPL (n=5) vs. 100 dB SPL (n=5) | 0.9997 | ns |  |  |
| 95 dB SPL (n=4) vs. 100 dB SPL (n=5) | 0.7723 | ns |  |  |

ns = not significant, **P<0.01.
