# Supplementary material for: Endolymphatic Hydrops is a Marker of Synaptopathy Following Traumatic Noise Exposure
Source: Front Cell Dev Biol. 2021 Nov 5;9:747870. doi: 10.3389/fcell.2021.747870 (PMC8602199; doi:10.3389/fcell.2021.747870)
Supplement: Supplementary file 12 [file Table6.DOCX]

Supplementary Table 6

| **Fig. 5E** |  |  |  |  |
| --- | --- | --- | --- | --- |
|  | W value | P value | Passed normality test (alpha=0.05)? |  |
| Shapiro-Wilk test for normality | 0.9639 | 0.2824 | Yes |  |
|  |  |  |  |  |
| Repeated measures two-way ANOVA | Sum of Squares | F value | P value | Significance |
| Interaction | 1.977 | 4.484 | 0.0075 | ** |
| Time | 3.205 | 21.80 | 0.0004 | *** |
| Treatment | 29.47 | 50.91 | <0.0001 | **** |
| Repeated measures | 1.544 | 2.626 | 0.0477 | * |
| Residual | 1.176 |  |  |  |
|  |  |  |  |  |
| Tukey's multiple comparisons test |  |  |  |  |
| 3 h | P value | Significance |  |  |
| Control (n=3) vs. 100 dB SPL, No treatment (n=3) | 0.1959 | ns |  |  |
| Control (n=3) vs. 100 dB SPL, 6000 mOsm/kg (n=3) | 0.1602 | ns |  |  |
| Control (n=3) vs. 100 dB SPL, 307 mOsm/kg (n=3) | 0.0017 | ** |  |  |
| 100 dB SPL, No treatment (n=3) vs. 100 dB SPL, 6000 mOsm/kg (n=3) | 0.525 | ns |  |  |
| 100 dB SPL, No treatment (n=3) vs. 100 dB SPL, 307 mOsm/kg (n=3) | 0.9003 | ns |  |  |
| 100 dB SPL, 6000 mOsm/kg (n=3) vs. 100 dB SPL, 307 mOsm/kg (n=3) | 0.0584 | ns |  |  |
|  |  |  |  |  |
| 5 h |  |  |  |  |
| Control (n=3) vs. 100 dB SPL, No treatment (n=3) | 0.0403 | * |  |  |
| Control (n=3) vs. 100 dB SPL, 6000 mOsm/kg (n=3) | 0.0100 | * |  |  |
| Control (n=3) vs. 100 dB SPL, 307 mOsm/kg (n=3) | 0.0059 | ** |  |  |
| 100 dB SPL, No treatment (n=3) vs. 100 dB SPL, 6000 mOsm/kg (n=3) | 0.1137 | ns |  |  |
| 100 dB SPL, No treatment (n=3) vs. 100 dB SPL, 307 mOsm/kg (n=3) | 0.9901 | ns |  |  |
| 100 dB SPL, 6000 mOsm/kg (n=3) vs. 100 dB SPL, 307 mOsm/kg (n=3) | 0.0090 | ** |  |  |
|  |  |  |  |  |
| 7 h |  |  |  |  |
| Control (n=3) vs. 100 dB SPL, No treatment (n=3) | 0.0004 | *** |  |  |
| Control (n=3) vs. 100 dB SPL, 6000 mOsm/kg (n=3) | 0.0057 | ** |  |  |
| Control (n=3) vs. 100 dB SPL, 307 mOsm/kg (n=3) | 0.0011 | ** |  |  |
| 100 dB SPL, No treatment (n=3) vs. 100 dB SPL, 6000 mOsm/kg (n=3) | 0.0047 | ** |  |  |
| 100 dB SPL, No treatment (n=3) vs. 100 dB SPL, 307 mOsm/kg (n=3) | 0.4395 | ns |  |  |
| 100 dB SPL, 6000 mOsm/kg (n=3) vs. 100 dB SPL, 307 mOsm/kg (n=3) | 0.0207 | * |  |  |

ns = not significant, *P<0.05, **P<0.01, ***P<0.001, ****P<0.0001.
